# Integrating socioeconomic context with multimodal EEG data for improved ADHD risk screening

**DOI:** 10.1371/journal.pone.0357213

**Published:** 2026-09-01

**Authors:** Sadikul Haque Sadi, Md. Arman Hossain, Md. Nurul Ahad Tawhid

**Affiliations:** 1 Institute of Information Technology, University of Dhaka, Dhaka, Bangladesh; 2 Department of Computer Science and Engineering, East West University, Dhaka, Bangladesh; Mae Fah Luang University School of Anti Aging and Regenerative Medicine, THAILAND

## Abstract

Attention-deficit/hyperactivity disorder (ADHD) affects millions globally, yet current diagnostic approaches rely on subjective behavioral assessments without objective neurophysiological markers. While machine learning on electroencephalogram (EEG) data shows promise for automated ADHD risk screening, current methods focus only on brain signals and ignore socioeconomic factors that strongly affect neurodevelopment and ADHD risk. We introduce a novel multimodal deep learning architecture integrating three complementary streams: temporal EEG dynamics via one-dimensional convolutional-recurrent networks, spectro-temporal patterns via two-dimensional convolutional networks with spatial and channel attention, and socioeconomic context via feedforward processing, combined through an attention-based fusion mechanism. Using the Cognitive Electrophysiology in Socioeconomic Context dataset, we evaluate performance across four cognitive tasks with 5-fold stratified cross-validation, ablation studies and benchmarking against a state-of-the-art EEG classification model. Under epoch-level cross-validation, the multimodal approach outperforms EEG-only baselines across all four tasks, achieving accuracy improvements of 2.1–5.9% and sensitivity gains up to 12.2%, with strong positive-class F1-scores (96.4–99.8%). Results showed higher epoch-level performance when socioeconomic context was incorporated alongside neurophysiological signals, a pattern that held across diverse cognitive paradigms. Leave-One-Subject-Out Cross-Validation across all four tasks yielded accuracy of 0.86–0.91 for the EEG-only model and 0.92–0.96 for the multimodal model, with sensitivity of 0.71–0.96 and specificity of 0.95–1.00 for the multimodal model. These subject-independent estimates are more modest than the epoch-level figures and McNemar’s test on paired predictions did not reach significance on any task. The EEG backbone, evaluated without modification on an independent paediatric dataset, also achieved 80.4% subject-independent accuracy, outperforming the prior benchmark. Labels derive from a validated self-report screening instrument rather than clinical diagnosis; this model should be understood as a proof-of-concept for ADHD risk screening, not a diagnostic tool. This work suggests the feasibility of context-aware ADHD risk screening that accounts for environmental influences on neurodevelopment alongside neurophysiological signals.

## Introduction

Attention-deficit/hyperactivity disorder (ADHD) is one of the most prevalent neurodevelopmental disorders, with global estimates suggesting it affects roughly 5–7% of children and approximately 2–5% of adults worldwide [1,2]. It is characterized by ongoing patterns of inattention, hyperactivity and impulsivity [3] that emerge during preschool years and intensify in school-age environments [4,5]. These symptoms negatively impact academic, personal and social functioning, with effects persisting into adulthood [6,7]. While prevalence estimates vary by region and diagnostic criteria, a 2023 umbrella review estimated the global rate in children and adolescents at 8.0% [8], reflecting both genuine cross-national differences and variation in screening and diagnostic practices.

Current diagnostic practices rely heavily on subjective behavioral assessments and clinical interviews, which are susceptible to reporting biases, cultural variations and lack objective neurophysiological markers [9]. This often results in delayed or missed diagnoses, particularly where specialized expertise is limited. Electroencephalography (EEG) offers a promising, low-cost avenue for developing objective ADHD biomarkers by directly measuring neural activity with high temporal resolution [10–12].

Recent deep learning advances have demonstrated automated EEG-based ADHD classification [13,14]; however, these approaches focus exclusively on brain signals and overlook socioeconomic context that profoundly influences neurodevelopment and ADHD risk. Extensive neuroscience research establishes that socioeconomic status (SES) including parental education, household income, food security and neighborhood quality significantly shapes brain development and cognitive function throughout life [15,16], with lasting effects into adulthood [9]. Socioeconomic adversity associates with increased attention and executive function deficits overlapping with ADHD symptomatology [17], suggesting SES factors may modulate neural signatures captured by EEG.

Despite well documented relationships between SES and both brain function and ADHD risk, machine learning approaches have not systematically integrated socioeconomic information with neurophysiological data. While neuroscience research has established measurable associations between socioeconomic indicators and electrophysiological responses [18], this knowledge has not been translated into computational models for clinical classification. Additionally, prior EEG-based studies typically rely on single data representations, temporal or spectral features, potentially missing complementary information [19].

In this study, we introduce a multimodal deep learning architecture integrating three complementary streams: (1) temporal features from raw EEG via 1D convolutional-recurrent networks, (2) spectro-temporal features from spectrograms via 2D convolutional neural networks (CNNs) with attention and (3) socioeconomic features via feedforward processing. Using the Cognitive Electrophysiology in Socioeconomic Context dataset [18], which includes EEG data from 127 young adults across four cognitive tasks along with detailed socioeconomic information, this study aims to address two key gaps in the existing research. First, although neuroscience has clearly shown that socioeconomic factors influence the neural activity captured by EEG, no previous studies have examined whether incorporating this knowledge can directly improve the accuracy of automated ADHD classification. We examine whether combining detailed socioeconomic information covering childhood family background and adult living conditions with multimodal EEG data can improve classification accuracy compared to relying on EEG signals alone.

Second, different cognitive tasks measure different aspects of attention and executive control. We therefore test whether the benefits of adding socioeconomic information are limited to specific tasks or reflect a consistent, generalizable effect. We evaluate our approach across four diverse cognitive paradigms: passive attention processing (auditory oddball), active target detection (visual oddball), response inhibition (flanker task) and visual search efficiency. This multi-task evaluation allows us to assess whether socioeconomic context provides consistent discriminative value across different cognitive demands or if its utility depends on the specific attentional processes being assessed.

We hypothesized that (H1) a multimodal model incorporating both EEG and SES features would achieve higher ADHD risk screening accuracy than an EEG-only baseline, and (H2) this benefit would hold across multiple cognitive paradigms rather than being limited to a single task; both hypotheses concern the SES effect and are tested exclusively on our primary dataset, since the external paediatric dataset used later in this study contains no socioeconomic variables. We separately hypothesized that (H3) the EEG-only backbone, applied without modification to that independent paediatric dataset, would achieve subject-independent accuracy at least comparable to the prior subject-independent benchmark reported for it; this hypothesis concerns architectural transfer alone and carries no SES claim.

## Related work

This section reviews methods for EEG-based ADHD detection, the influence of socioeconomic factors on neurodevelopment and approaches to multimodal integration.

### EEG-based ADHD classification

#### Traditional machine learning approaches.

Early EEG-based ADHD classification relied on manually engineered features (that is, domain-specific signal properties such as power spectra and entropy measures defined by experts rather than learned from data) and conventional machine learning algorithms [12,20]. As computational approaches matured, researchers employed statistical measures, power spectral densities across frequency bands [11] and nonlinear dynamics features such as entropy and fractal dimensions [21–23].

Feature selection techniques included statistical hypothesis testing and LASSO (Least Absolute Shrinkage and Selection Operator) regularization [24]. Altinkaynak et al. [25] showed that integrating time-domain Event-Related Potential features with frequency-domain characteristics achieved 91.3% binary classification accuracy on a small pediatric dataset using a support vector machine with leave-one-out cross-validation, suggesting multimodal feature representations within EEG could enhance classification performance.

However, traditional approaches faced fundamental limitations: dependence on domain expertise for feature definition, potential information loss through dimensionality reduction and inability to capture complex hierarchical patterns [12]. Reported classification accuracies varied widely (70–95%) depending on feature engineering choices, dataset characteristics and evaluation protocols [20]. It is worth noting that many of these studies relied on relatively small, demographically specific samples, which may limit how broadly their findings can be applied.

More recently, Chandela et al. [26] proposed a unified approach combining successive multivariate variational mode decomposition (SMVMD) with K-nearest neighbor classification for detecting multiple neurodevelopmental disorders in children, achieving 99.17% accuracy for ADHD classification. While such methods demonstrate high performance through carefully engineered features, they remain constrained by the need for domain expertise and may not fully capture hierarchical representations that deep learning can automatically discover.

#### Deep learning advances.

Deep learning transformed EEG analysis by enabling end-to-end feature learning. Researchers have successfully applied CNNs to spectro-temporal EEG representations. Dubreuil-Vall et al. [19] applied CNNs to event-related spectral EEG for ADHD classification, while Tawhid et al. [27] demonstrated that spectrogram-based approaches could achieve high accuracy (exceeding 95–99%) for autism spectrum disorder, establishing the viability of treating EEG spectrograms as images for CNN processing. Moghaddari et al. [28] developed a 13-layer CNN achieving 99.06% accuracy using segmented 4-second epochs.

Attention mechanisms have enhanced both performance and interpretability [29], with recent work proposing autoencoder-ResNet pipelines [13] and specialized architectures for EEG feature maps [14]. Tawhid et al. [30] demonstrated that generic CNN architectures can achieve robust cross-disorder classification across six neurological conditions including ADHD, suggesting shared neural signatures across neurodevelopmental disorders.

Most recently, Hossain and Tawhid [31] extended the spectrogram-CNN framework to schizophrenia detection, achieving 98.31–99.82% accuracy across two public EEG datasets and demonstrating that STFT-derived mel-spectrogram representations retain high discriminative power even when evaluated through brain-lobe-specific channel subsets, further reinforcing the viability of spectro-temporal image inputs for EEG-based psychiatric classification.

Methodological variations substantially impact classification outcomes [32], with reported accuracies ranging from 76–99% [33,34] reflecting algorithmic differences, dataset variations and evaluation protocols. Despite these advances, recent approaches share two critical limitations: (1) they process neurophysiological data isolated from socioeconomic context, despite established neuroscience showing environmental influences on brain development [16] and (2) most rely on single EEG representations (either temporal or spectral) rather than exploiting their complementary nature. Even in cases where EEG-only baselines approach high accuracy, there are reasons to expect SES integration to add value. Screening instruments such as the ASRS capture symptom burden shaped by environmental conditions, meaning that brain signals alone may not fully account for the variance introduced by socioeconomic adversity. Modeling this context explicitly may therefore improve predictive accuracy by accounting for environmental sources of variance that brain signals alone do not capture.

### Socioeconomic context in neurodevelopment

While computational approaches have focused exclusively on brain signals, developmental neuroscience has established that socioeconomic status profoundly shapes brain structure and function [15,16]. Early-life SES influences cognitive development in domains central to ADHD symptomatology, particularly executive function and attention [15,16,35–37]. Electrophysiological research shows SES affects event-related potential components during cognitive tasks [18], with effects persisting into adulthood.

Epidemiological studies consistently link socioeconomic adversity to elevated ADHD prevalence [17]. Despite robust evidence connecting SES to both brain function and ADHD risk, this knowledge has not been translated into computational models for clinical classification. It is also important to acknowledge that ADHD has a strong heritable component [38], which means low SES alone is not predictive of the disorder; rather, socioeconomic context likely interacts with genetic predisposition to modulate symptom expression and neural signatures. Furthermore, access to formal clinical diagnosis is itself SES-stratified: individuals from lower-income backgrounds are less likely to receive a diagnosis even when symptom burden is comparable [17], which means ASRS-based screening labels may themselves carry socioeconomic gradients that a model must account for rather than ignore.

### Multimodal learning and research gap

Multimodal learning integrates heterogeneous data types through fusion strategies including early concatenation, late combination and attention-based weighting [39]. In broader healthcare applications, multimodal approaches have successfully combined medical imaging with clinical variables. Within EEG analysis specifically, recent state-of-the-art methods in related affective and neurological domains have begun demonstrating the critical value of contextual data. For instance, demographic factors have been shown to significantly impact the generalizability of EEG-based emotion recognition models [40] and demographic attention mechanisms have been successfully employed to improve EEG-based depression detection [41]. Furthermore, compact multilayer perceptrons (MLPs) have been effectively used to fuse clinical and demographic tabular data with complex modalities to predict post-stroke cognitive decline [42]. Despite these advances in related fields, EEG-based ADHD classification has primarily focused on combining different neurophysiological measurements rather than integrating neural data with contextual socioeconomic information.

This reveals a critical disconnect: while neuroscience demonstrates socioeconomic shaping of neural substrates, computational models treat brain signals in contextual isolation. Our work bridges this gap by testing whether SES features improve ADHD classification when integrated with EEG data through attention-based fusion of temporal dynamics, spectro-temporal patterns and socioeconomic context.

## Materials and methods

In this section, we outline the approach we took for ADHD risk screening, including a breakdown of the dataset, how we preprocessed the data, the architecture of our model and our experimental setup.

### Dataset description

This study utilizes the “Cognitive Electrophysiology in Socioeconomic Context in Adulthood” dataset [18] which is publicly available on OpenNeuro at accession ds005863 (https://openneuro.org/datasets/ds005863). The dataset was collected at the University of Florida (USA) and investigates relationships between cognitive electrophysiology, socioeconomic status and ADHD symptomatology in young adults.

The dataset includes 127 young adults (mean age: 21.37±2.60 years; 57.5% female) recruited at the University of Florida (USA) from diverse socioeconomic backgrounds [18]. We used data from four ERP CORE cognitive tasks. The *auditory oddball* task required participants to press a button whenever an infrequent target tone appeared among frequent standard tones, probing passive auditory attention and P3 generation. The *visual oddball* task followed the same logic but used visual stimuli, requiring detection of an infrequent target shape. The *flanker* task measured response inhibition: participants indicated the direction of a central arrow while ignoring flanking arrows that were either congruent or incongruent with the target. The *visual search* task assessed attentional selection by requiring participants to find a target letter among distractor letters. Full task specifications are available in the original dataset paper [18]. Available participant counts per task were: auditory oddball (114), visual oddball (99), flanker (73) and visual search (72). Not all participants completed every task, as the dataset was collected across multiple ongoing studies [18]. Our inclusion criteria required both complete EEG recordings for a given task and a valid ADHD screening label from the ASRS-v1.1. After applying these criteria, final sample sizes were: auditory oddball (n = 95), visual oddball (n = 84), flanker (n = 60) and visual search (n = 59). The reduction from the total pool of 127 therefore reflects both task non-completion and missing screening data, rather than EEG data quality failure alone.

**Fig 1:**
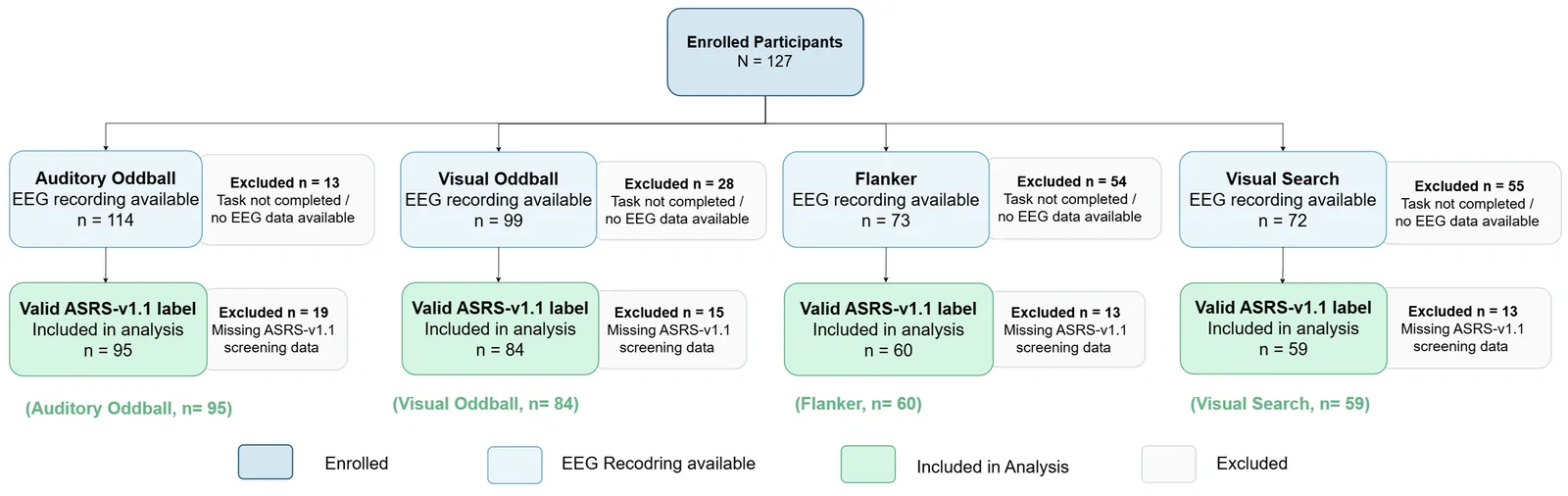
Participant flow diagram across four cognitive tasks. From an enrolled pool of *N* = 127 participants, task-specific samples reflect two sequential inclusion criteria: **(1)** availability of a complete EEG recording for the given task and **(2)** a valid ASRS-v1.1 screening label. Final analysed samples: Auditory Oddball (*n* = 95); Visual Oddball (*n* = 84); Flanker (*n* = 60); Visual Search (*n* = 59).

Fig 1 illustrates the participant flow and exclusion criteria for each cognitive task.

ADHD symptomatology was assessed using the WHO Adult ADHD Self-Report Scale (ASRS-v1.1). Participants were classified as “ADHD-screened-positive” if they endorsed ≥4 of the first six items at the validated screening threshold. Per the dataset authors’ own characterization, these labels identify individuals with “symptoms consistent with ADHD” rather than clinically confirmed cases [18]. **Important caveat:** The ASRS-v1.1 is a validated screening instrument, not a diagnostic tool; all labels in this study reflect self-reported screening status rather than confirmed clinical diagnoses and the model should be understood as a screening-support tool for identifying individuals at elevated ADHD risk. The socioeconomic features comprised 17 variables spanning four key areas. Subjective social status in childhood was captured using an adapted MacArthur Scale, where participants retrospectively ranked their family’s standing at age 10 on a 10-step ladder. Educational attainment was captured with two variables: the participant’s own highest completed education level and the highest education level completed by a parent or legal guardian. To evaluate current food security over the past 12 months, we used the six-item short form of the USDA Household Food Security Survey Module. Lastly, current home and neighborhood characteristics were recorded using 8 items adapted from the Survey of Income and Program Participation (SIPP), detailing environmental factors such as structural housing issues, street noise and perceived neighborhood safety.

EEG data were recorded at 500 Hz using a Brain Products actiCHamp Plus amplifier with a 32-channel actiCAP slim active electrode cap (Brain Products GmbH, Gilching, Germany). Electrodes were mounted according to the extended 10–20 system, with FPz serving as the ground and Cz as the online reference. Six channels were excluded from analysis: five repurposed as non-scalp sensors, specifically two mastoid electrodes (LM, RM) used for offline re-referencing and three electrooculogram (EOG) electrodes (HEL, VER, HER) used for artifact detection, along with the online reference (Cz), which was removed following re-referencing. Notably, the T8 cap position was occupied by the right mastoid electrode (RM) and is therefore absent from the scalp montage. This yielded **26 scalp EEG electrodes**: Fp1, Fp2, F7, F3, Fz, F4, F8, FC5, FC1, FC2, FC6, T7, C3, C4, CP5, CP1, CP2, CP6, P7, P3, Pz, P4, P8, O1, Oz and O2, spanning prefrontal, frontal, fronto-central, central, centro-parietal, parietal and occipital regions. The full electrode configuration is illustrated in Fig 1 of [18].

### Data preprocessing and feature engineering

To prepare the data for our multimodal architecture, we implemented distinct preprocessing pipelines for the raw EEG signals, their time-frequency representations and the socioeconomic features.

#### EEG signal preprocessing.

We preprocessed the raw EEG recordings using MNE-Python. We applied a bandpass filter between 0.5 and 40 Hz to remove slow cortical drifts and high-frequency muscle artifacts. To balance computational efficiency with signal fidelity, we downsampled the recordings from 500 to 256 Hz, a sampling rate widely adopted in EEG-based deep learning as it fully preserves all neural frequency content relevant to our analysis [43]. These cleaned signals then proceeded to the segmentation phase.

#### EEG signal segmentation.

We segmented the continuous, cleaned EEG recordings from each cognitive task into fixed-length, 4.0-second non-overlapping epochs. We acknowledge that the four tasks are ERP paradigms from the ERP CORE battery, conventionally analysed using stimulus-locked epochs. Our continuous segmentation follows an established convention in deep learning EEG classification rather than ERP component analysis, where fixed-length windows are preferred over stimulus-locked epochs.

Unlike trial averaging, convolutional classifiers extract locally invariant features across the input window without requiring a fixed stimulus reference point. More importantly, ADHD-discriminative neural patterns are not limited to sharp ERP peaks: theta/beta power differences, sustained attention fluctuations, and slow cortical activity manifest continuously throughout the recording [12] and a 4-second window captures these more completely than short event-locked windows that would truncate them. This segmentation approach is established in deep learning EEG pipelines [28,44] and Tawhid et al. [30] confirmed 4-second epochs as a robust configuration through systematic ablation across 1–4 second lengths. Most directly, applying this identical epoch structure without modification to the Nasrabadi visual attention ERP task [45] achieved 97.5% epoch-level and 80.4% subject-independent accuracy (Table 8), confirming its effectiveness for ERP-based deep learning classification. Given our 256 Hz sampling rate, each epoch corresponds to 1,024 time points, yielding input tensors shaped 26 channels × 1,024 time points per sample.

#### 1D temporal feature preparation.

To prepare the features for our 1D CNN-recurrent neural network (RNN) stream, we applied a global normalization scheme to the segmented epochs. Standard normalization techniques (using mean and standard deviation) are highly sensitive to the exact kind of outliers and artifacts that frequently appear in EEG data [46]. To ensure our normalization is robust against anomalies, we utilized the Median Absolute Deviation (MAD), a well-established statistical measure resilient to extreme values [47,48]. This MAD-based normalization was applied on a per-epoch basis by computing a single scalar across all channels and time points within each epoch, after which we clipped the resulting values to a range of [−10, 10] to safely eliminate any residual outliers.

#### 2D spectro-temporal feature generation.

For the 2D CNN stream, we converted each 4-second EEG epoch into a multi-channel time-frequency representation. We computed a spectrogram for each of the 26 channels using the Short-Time Fourier Transform (STFT) with a Hann window. Key parameters for the spectrogram computation were an nperseg of 256, an noverlap of 128 and an nfft of 512. The resulting power spectral density was log-transformed ($10\log_{10}(\text{Sxx})$) and normalized to a [0, 1] range by clipping based on the 5th and 95th percentiles. Finally, each channel’s spectrogram was resized to a fixed dimension of 64×64 pixels, yielding a final input tensor of shape 26×64×64 for each sample.

#### SES feature processing.

We processed 17 raw SES variables into a 40-dimensional input vector via one-hot encoding of categorical variables and direct retention of the single continuous variable (Table 1). The variables cover four constructs: subjective social standing (1 continuous item), food security (6 items from the USDA Food Security Scale [18]), neighbourhood and community conditions (8 items) and educational attainment of the participant and household adults (2 grouped variables). Categorical variables were one-hot encoded, the single continuous variable was standardized using a standard scaler and missing values were handled via median imputation for the continuous column and mode imputation for categorical columns, both well-established, outlier-robust strategies for socioeconomic survey data [49,50].

**Table 1:** SES feature encoding: 17 raw variables to 40-dimensional input vector. Categorical variables are expanded via one-hot encoding; the single continuous variable is retained as-is.

Variable(s)	Construct	Type	Dims
Subjective_SES	Self-rated social standing	Continuous	1
fs1, fs2, fs4	Food security items (3-category)	Categorical	9
fs3, fs5, fs6	Food security items (2-category)	Categorical	6
hnc1a–hnc1d	Neighbourhood conditions (binary)	Categorical	8
hnc2a, hnc2b	Neighbourhood conditions (binary)	Categorical	4
hnc3	Neighbourhood condition (binary)	Categorical	2
hnc4	Neighbourhood condition (4-category)	Categorical	4
Highest_Edu_Grouped	Participant education level	Categorical	3
Highest_Adult_Edu_Grouped	Household adult education level	Categorical	3
**Total (17 variables)**			**40**

### Proposed multimodal fusion architecture

**Fig 2:**
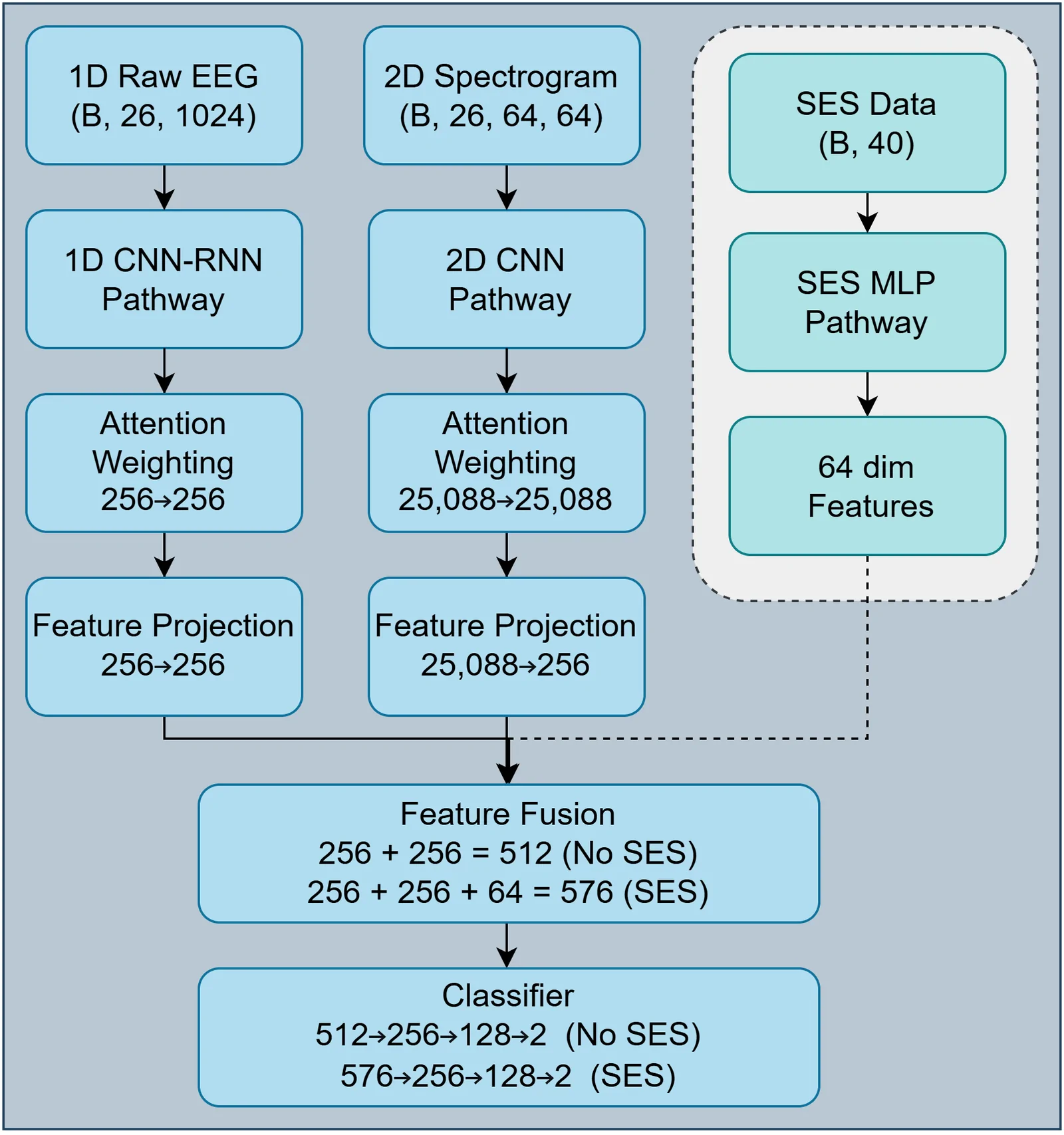
The proposed multimodal fusion architecture. Three parallel streams process heterogeneous inputs simultaneously: a 1D CNN-RNN pathway extracts temporal dynamics from raw EEG (producing a 256-dimensional vector), a 2D CNN pathway with integrated spatial and channel attention extracts spectro-temporal patterns from log-spectrograms (producing a 25,088-dimensional vector projected to 256 dimensions) and a feedforward SES pathway encodes socioeconomic context (producing a 64-dimensional embedding). The three representations are concatenated into a 576-dimensional multimodal vector and passed to a three-layer classification head. Solid lines indicate components present in both With-SES and Without-SES configurations; dashed lines indicate the optional SES stream used only in the full multimodal model.

The design of our multimodal fusion architecture is directly motivated by the limitations identified in the literature (as discussed in the Related work section). To address the prevalent use of single-modality EEG representations, our model incorporates two specialized, parallel streams to capture complementary neurophysiological information: a 1D convolutional-recurrent pathway to model raw temporal dynamics and a 2D convolutional pathway to learn from spectro-temporal image representations. This dual-stream approach is inspired by work showing the distinct benefits of both temporal and frequency-domain features [25]. To bridge the critical gap between computational models and neuroscientific findings, a third feedforward stream is integrated to process socioeconomic context. Finally, informed by successful general applications in multimodal learning [39] and recent evidence showing that attention mechanisms effectively integrate demographic context with CNN-processed EEG signals [41], we employ an attention-based fusion mechanism. We developed a novel deep learning architecture that integrates three parallel pathways to process raw EEG, spectrograms and socioeconomic data simultaneously. Rather than merging these modalities using rigid, fixed combination rules, this design allows the model to adaptively learn the dynamic, sample-specific importance of each data stream. A diagram of the proposed model is shown in Fig 2.

#### 1D temporal pathway (CNN-RNN).

This stream processes the raw 1D EEG signals. It begins with a convolutional layer (kernel size 7, stride 2) followed by a series of residual blocks. These blocks progressively extract hierarchical temporal features. The output from the convolutional layers is then fed into a 2-layer bidirectional Gated Recurrent Unit (GRU) with a hidden dimension of 128. The GRU captures long-range temporal dependencies in the signal. Finally, global average pooling is applied to the GRU’s output to produce a 256-dimensional temporal feature vector.

The complete layer-by-layer specifications of the 1D temporal stream are detailed in Table 2. The progressive downsampling through strided convolutions (stride 2) reduces the temporal resolution from 1,024–128 time steps while increasing feature depth from 26 to 256 channels. The residual connections in both blocks facilitate gradient flow during training and enable the learning of hierarchical temporal representations.

**Table 2:** 1D temporal stream architecture for raw EEG.

Layer	Output	Kernel	Stride	Details
Input	(26, 1024)	–	–	26 channels, 4s epoch
Conv1D + BN + ReLU	(64, 512)	7	2	Feature Extraction
*ResBlock 1 (64* → *128)*				
Conv1D + BN + ReLU	(128, 256)	3	2	Downsampling
Conv1D + BN	(128, 256)	3	1	Processing
Skip connection	(128, 256)	1	2	Residual Link
*ResBlock 2 (128* → *256)*				
Conv1D + BN + ReLU	(256, 128)	3	2	Downsampling
Conv1D + BN	(256, 128)	3	1	Processing
Skip connection	(256, 128)	1	2	Residual Link
BiGRU (2×128 bidir)	(128, 256)	–	–	Temporal Modeling
GlobalAvgPool1D	(256)	–	–	Vectorization

Total trainable parameters: 1,017,792

#### 2D spectro-temporal pathway (2D CNN).

This pathway analyzes the multi-channel spectrograms. It employs a deep 2D CNN designed to capture both spatial (across channels) and spectro-temporal patterns. The architecture includes integrated spatial and channel-wise attention mechanisms, allowing the model to focus on the most informative frequency bands, time segments and channel locations. An adaptive average pooling layer reduces the feature map to a fixed size of 512×7×7, which is then flattened to produce a 25,088-dimensional spectral feature vector.

Full layer-by-layer specifications are provided in Table 3. The key design principle is progressive spatial compression: four max-pooling stages reduce the input from 64×64 to 4×4 while channel depth grows from 64 to 512, concentrating discriminative spectro-temporal information into a compact feature map on which the dual attention mechanisms then operate. Adaptive average pooling then standardises this to a fixed 512×7×7 representation regardless of input length, yielding the 25,088-dimensional vector passed to the fusion stage.

**Table 3:** 2D spectro-temporal stream architecture for spectrograms.

Layer	Output Shape	Kernel	Stride	Details
Input	(26, 64, 64)	–	–	Log-transformed Spectrogram
Conv2D + BN	(64, 64, 64)	5×5	1	ReLU Activation
MaxPool2D	(64, 32, 32)	2×2	2	–
Conv2D + BN	(128, 32, 32)	1×1	1	ReLU Activation
Conv2D + BN	(256, 32, 32)	3×3	1	ReLU Activation
MaxPool2D + Dropout	(256, 16, 16)	2×2	2	Drop rate: 0.25
Conv2D + BN	(256, 16, 16)	3×3	1	ReLU Activation
MaxPool2D	(256, 8, 8)	2×2	2	–
Conv2D + BN	(512, 8, 8)	3×3	1	ReLU Activation
MaxPool2D + Dropout	(512, 4, 4)	2×2	2	Drop rate: 0.25
*Spatial Attention*				
Conv2D + ReLU	(256, 4, 4)	1×1	1	Attention Weights
Conv2D + Sigmoid	(512, 4, 4)	1×1	1	Mask Generation
Multiply	(512, 4, 4)	–	–	Element-wise Application
*Channel Attention*				
GlobalAvgPool	(512)	–	–	Squeeze Operation
Linear + ReLU	(256)	–	–	Excitation (Reduction)
Linear + Sigmoid	(512)	–	–	Scaling Weights
Multiply	(512, 4, 4)	–	–	Channel-wise Scaling
AdaptiveAvgPool2D	(512, 7, 7)	–	–	Feature Map Normalization
Flatten	(25,088)	–	–	Vectorization

Total trainable parameters: 2,643,648.

#### SES pathway (feedforward network).

The SES pathway processes the 40-dimensional contextual feature vector through a compact two-layer feedforward network. The first layer expands the representation to 128 dimensions (${𝐖}_{1}\in {\mathbb{R}}^{40\times 128}$), followed by batch normalization, ReLU activation and dropout with a rate of 0.3 for regularization. The second layer compresses this to 64 dimensions (${𝐖}_{2}\in {\mathbb{R}}^{128\times 64}$), again followed by batch normalization, ReLU and dropout (0.3). This architecture yields a 64-dimensional embedding that encodes socioeconomic and clinical context with minimal parameters (13,888 trainable parameters, representing just 0.04% of the total model). This compact MLP design closely aligns with recent state-of-the-art multimodal frameworks. For example, Filippis and Foysal [42] utilized a similar dimensionality reduction strategy (compressing tabular clinical data through 64→32 hidden layers to output a 16-dimensional vector) for post-stroke cognitive decline prediction. In our architecture, the 128→64 projection serves a specific, parallel design goal: it provides essential contextual background without allowing the low-dimensional tabular data to overpower the massive, high-dimensional EEG representations during fusion.

#### Attention-based multimodal fusion.

**Fig 3:**
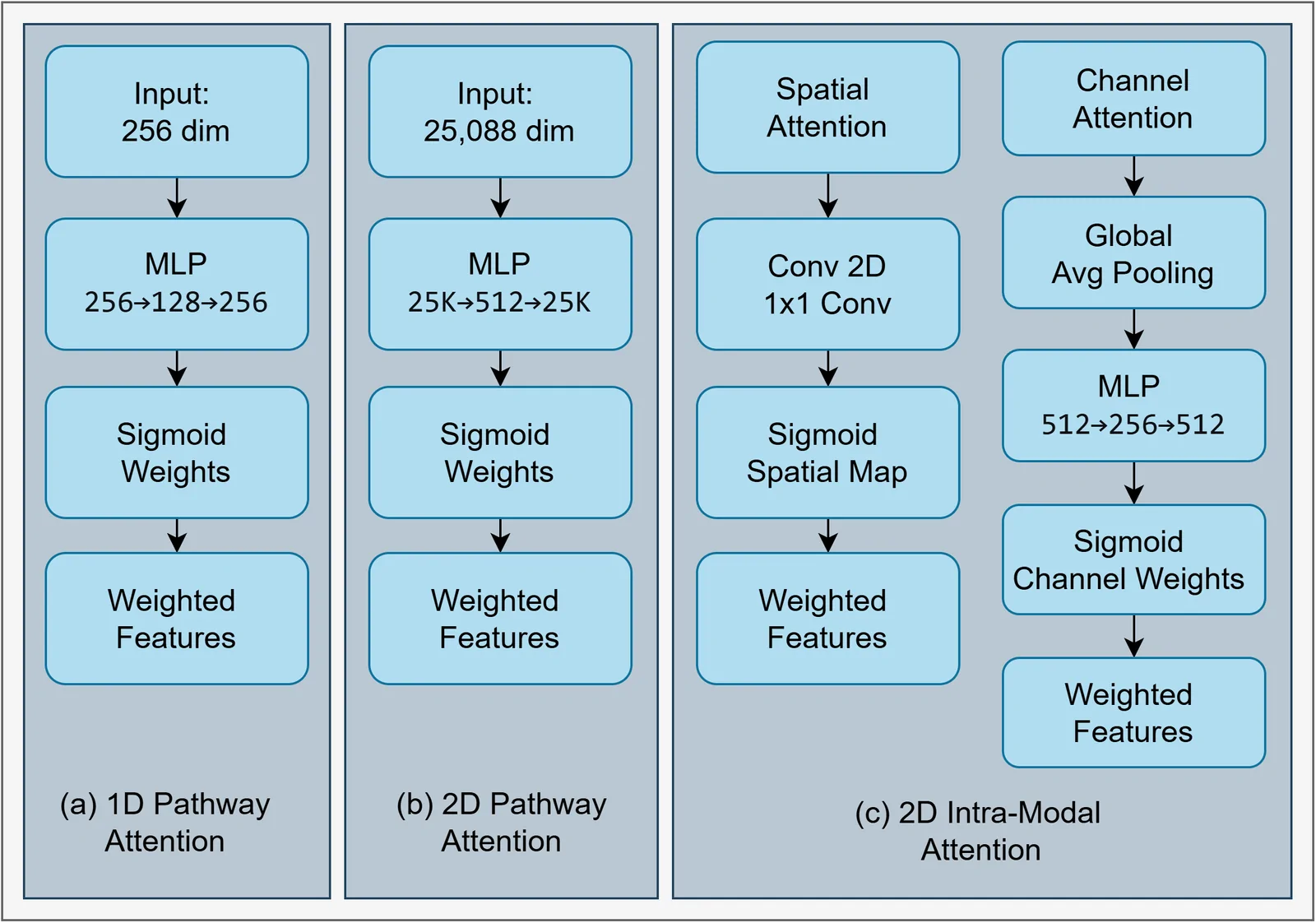
Overview of attention mechanisms. (a) 1D pathway uses MLP-based attention for temporal features, (b) 2D pathway employs bottleneck MLP for high-dimensional spectral features, (c) intra-modal attention combines spatial and channel mechanisms for spectrogram analysis.

To address our primary research question, we implemented sophisticated attention mechanisms (Fig 3) that dynamically weight feature importance.

1. **Attention weighting**: The 1D stream uses an MLP (256→128→256) with a sigmoid function to learn temporal pattern importance. The 2D stream employs a bottleneck MLP (25,088→512→25,088) to handle spectral feature compression. Additionally, intra-modal dual spatial (1×1 conv) and channel (global pool + MLP) attention is applied for spectrograms.
2. **Feature projection**: The attention-weighted EEG features are projected into a 256-dimensional common space.
3. **Concatenation**: The projected 1D (256), 2D (256) and SES (64) features are combined to form a single 576-dimensional representation.
4. **Classification**: A three-layer classifier (576→256→128→2) produces the final predictions.

The attention mechanisms operate at multiple scales within the architecture. For the 1D temporal stream, a multi-layer perceptron with a bottleneck architecture (256→128→256 followed by sigmoid activation) learns to weight the importance of different temporal features extracted by the CNN-RNN pathway. The 2D spectro-temporal stream employs a more aggressive bottleneck due to its high dimensionality (25,088→512→25,088), which efficiently learns which spectral features are most discriminative while managing computational complexity.

Additionally, the 2D stream incorporates intra-modal attention mechanisms that operate at earlier stages of feature extraction. Spatial attention (Fig 3c, detailed in Table 3) uses two successive 1×1 convolutional layers to generate attention weights for each spatial location in the 4×4 feature map, effectively identifying which time-frequency regions contain the most diagnostic information. Channel attention (also shown in Fig 3c) applies global average pooling followed by a two-layer MLP to produce weights for each of the 512 feature channels, allowing the model to emphasize channels that have learned to detect relevant EEG patterns. Both attention mechanisms use sigmoid activation to produce weights in the range [0, 1], which are then multiplied element-wise with the original feature maps.

Following attention weighting, the features from both EEG streams are projected into a common 256-dimensional subspace through learned linear transformations. This projection step is critical for balanced multimodal fusion, as it prevents the high-dimensional 2D features (25,088 dimensions) from dominating the concatenated representation. The projected 1D features (256 dimensions), projected 2D features (256 dimensions) and SES features (64 dimensions) are then concatenated to form the final 576-dimensional multimodal representation that captures complementary information from all three input modalities.

The parameter distribution across model components, summarized in Table 4, reveals that 89.3% of the model’s parameters are dedicated to the fusion module, with 71.4% specifically allocated to attention mechanisms and 18.0% to feature projections. This significant focus on learning adaptive fusion weights reflects the model’s core design principle: instead of independently extracting fixed features from each modality, it dynamically learns how to weight and combine multimodal information based on each sample’s unique characteristics. The SES processing network, despite its compact architecture (13,888 parameters, 0.04% of total), provides important contextual information that complements the EEG-derived features in the final representation.

**Table 4:** Parameter distribution across model components.

Component	Parameters	%
1D Temporal Stream	1,017,792	2.82
2D Spectro-Temporal Stream	2,643,648	7.32
SES Processing Network	13,888	0.04
*Fusion Module*		
Attention Mechanisms	25,781,632	71.36
Feature Projections	6,488,576	17.96
Subtotal (Fusion)	32,270,208	89.32
Classification Head	181,634	0.50
**Total Parameters**	**36,127,170**	**100.00**
**Trainable Parameters**	**36,127,170**	**100.00**

The final classification head consists of three fully connected layers with progressive dimensionality reduction: 576→256→128→2. Each hidden layer is followed by batch normalization, ReLU activation and dropout with a rate of 0.5 to prevent overfitting on the fused multimodal representation. The output layer produces logits for the two classes (ADHD vs. Control), which are converted to probabilities via softmax during inference. This classifier contains 181,634 parameters (0.50% of the total model), reflecting its design as a simple decision boundary learner that operates on the rich, attention-weighted, multimodal features rather than performing additional complex feature extraction.

### Experimental setup and training

The model was trained and evaluated using a rigorous protocol to ensure robust and generalizable results.

#### Validation strategies.

To comprehensively evaluate our model, we employed a two-tier validation strategy with clearly distinct purposes. First, for broad architectural benchmarking across all four tasks, we applied 5-fold Stratified K-Fold cross-validation at the *epoch level*: the dataset of EEG epochs is partitioned into five folds while preserving the ADHD/Control class ratio and every epoch is used for both training and independent testing across folds. Because this protocol operates on epochs rather than participants, epochs from the same individual may appear in both training and test partitions across different folds. Epoch-level cross-validation is standard practice in EEG deep learning, particularly when dataset sizes are small, since subject-level splits often leave too few training samples for reliable model comparison [30,51,52]. In this context, epoch-level evaluation provides a reproducible, high-statistical-power basis for comparing architectural configurations and should be read as such rather than as a claim of subject-independent generalization. The large number of test epochs per fold also explains the characteristically low fold-level variance seen in these results. Second, for subject-independent generalization (the clinically meaningful evaluation) we implemented a Leave-One-Subject-Out Cross-Validation (LOOCV) protocol. In LOOCV, the model is trained on all subjects except one, which is held out entirely for testing; this process repeats until every participant has served as the test subject exactly once. This protocol guarantees that no epoch from a test participant is ever seen during training, providing a strict and unambiguous estimate of real-world generalizability.

#### Ablation and baseline models.

To quantify the contribution of each architectural component, we conducted an ablation study evaluating the isolated 1D temporal stream, the isolated 2D spectro-temporal stream and a ‘Simple Fusion’ model (concatenation without attention). Furthermore, to establish a standardized baseline against existing literature, we implemented EEGNet [53], a widely recognized, highly compact convolutional neural network considered a state-of-the-art benchmark for robust EEG-based brain-computer interfaces. Since prior approaches often employ highly variable preprocessing and segmentation pipelines, EEGNet provides a stable, standardized external comparison for our target dataset.

#### Training and regularization.

We implemented our architecture in PyTorch and trained it on an NVIDIA Tesla T4 GPU. To manage the substantial computational footprint of the tri-stream architecture while ensuring robust convergence, our hyperparameter selection was guided by established best practices in deep learning and physiological signal processing, followed by targeted empirical tuning on our validation set. Models were trained for a maximum of 100 epochs, using an early stopping patience of 20 epochs based on the validation F1-score to prevent overtraining.

We optimized the network using AdamW [54], implementing component-specific learning rates (1D-stream: $1\times 10^{-4}$, 2D-stream: $5\times 10^{-5}$, fusion/classifier: $1-2\times 10^{-4}$) to account for the varying representational densities of the temporal, spatial and tabular pathways, coupled with a cosine annealing scheduler (*T*_0_ = 10, $T_{mult}=2$).

To ensure robust generalization and handle the inherent class imbalance of clinical datasets, we integrated several established regularization techniques: a Focal Loss function [55] (γ=2.0, α set to inverse class-frequency weights computed from each training fold) to down-weight easy majority-class examples and focus learning on harder minority-class cases, L2 regularization ($1\times 10^{-4}$) and gradient clipping (max norm 1.0). Furthermore, we employed strategic dropout combined with batch normalization throughout the network. Specifically, we adopted a 25% dropout rate (0.25) following the spatial pooling blocks in our 2D spectro-temporal stream—a regularization pattern previously validated for robust multi-disorder EEG classification [30]—alongside rates of 0.3 and 0.5 in the feedforward and classification heads to prevent overfitting on the fused representations.

### Evaluation metrics

To evaluate the model’s performance in distinguishing between ADHD and control participants, we used a comprehensive set of metrics calculated from the validation set of each fold which are commonly used in this field of study [56–59]. The primary metrics include:

- **Accuracy:** The overall proportion of correct classifications.
- **Weighted F1-Score:** The F1-score weighted by the support of each class, providing a balanced measure of performance on imbalanced datasets.
- **Sensitivity (Sens) / Recall:** The model’s ability to correctly identify true positive cases (ADHD).
- **Specificity (Spec):** The model’s ability to correctly identify true negative cases (Control).

We also report the F1-score specifically for the ADHD class to assess performance on the minority class of interest, alongside global Sensitivity and Specificity. The final 5-fold cross-validation results are presented as the mean and standard deviation across the folds.

#### Comparative conditions.

- **With SES**: Full multimodal architecture with socioeconomic context
- **Without SES**: Same architecture excluding the SES processing stream
- **Task-specific**: Separate models for each cognitive task (Flanker, Auditory Oddball, Visual Oddball, Visual Search)

## Experimental results

In this section, we present the classification performance of our multimodal fusion architecture across the four EEG cognitive tasks under two configurations: EEG-only (1D temporal and 2D spectro-temporal streams) and the full multimodal model integrating EEG with socioeconomic context. Results are reported under a two-tier validation framework with explicitly different interpretations. Epoch-level 5-fold stratified cross-validation serves as the architectural benchmarking tier: it operates on the full epoch pool, enabling high-powered comparison of model configurations but not constituting a subject-independent generalization claim. LOOCV serves as the subject-independent generalization tier: it guarantees complete participant separation between training and test sets and is the primary basis for any claim about real-world performance.

### Task-specific dataset characteristics

Table 5 summarizes the dataset characteristics after preprocessing. The varying segment counts reflect different trial structures, with the visual search and flanker tasks yielding more segments due to their shorter trial durations.

**Table 5:** Dataset characteristics after preprocessing.

Task	Participants	Segments	ADHD	Control
Auditory Oddball	95	5,944	1,938	4,006
Visual Oddball	84	7,747	2,326	5,421
Visual Search	59	10,119	2,986	7,133
Flanker	60	12,626	3,549	9,077

### Architectural ablation and SOTA comparison

Table 6 shows the ablation results across all four cognitive tasks. The proposed model leads on accuracy and ADHD F1-score in every task and takes the top spot on most other metrics too. The 2D stream on its own was the weakest configuration across all four tasks, which confirms that spectrogram features alone are not enough; they need the 1D temporal stream to provide reliable classification. Simple fusion came close to the proposed model in some tasks but fell short on the metrics that matter most for ADHD screening, particularly ADHD F1-score and sensitivity.

**Table 6:** Ablation and baseline comparison across all four cognitive tasks. Bold values mark the best result per metric within each task. Task abbreviations: AO = Auditory Oddball; VO = Visual Oddball; VS = Visual Search; FL = Flanker. Model abbreviations: 1D = 1D Stream Only; 2D = 2D Stream Only; S.Fus = Simple Fusion; EEGNet = EEGNet (SOTA); Prop. = Proposed model. W. F1 = weighted F1-score; ADHD F1 = ADHD-class F1-score; AUC = area under the ROC curve. All values are mean ± std over 5 folds.

Task	Model	Accuracy	W. F1	ADHD F1	Sensitivity	Specificity	AUC
**AO**	1D	0.900±0.011	0.900±0.011	0.851±0.016	0.840±0.018	0.930±0.015	0.901±0.016
	2D	0.833±0.023	0.833±0.021	0.752±0.026	0.774±0.061	0.861±0.052	0.847±0.025
	S.Fus	0.902±0.008	0.902±0.008	0.861±0.010	0.859±0.016	0.931±0.015	0.904±0.009
	EEGNet	0.906±0.003	0.906±0.003	0.868±0.005	0.887±0.029	0.930±0.015	0.939±0.007
	**Prop.**	0.917±0.005	0.916±0.004	0.870±0.004	0.851±0.028	0.949±0.019	0.935±0.010
**VO**	1D	0.946±0.007	0.946±0.008	0.909±0.013	0.900±0.020	0.966±0.008	0.920±0.006
	2D	0.900±0.015	0.902±0.015	0.842±0.022	0.883±0.042	0.908±0.029	0.902±0.008
	S.Fus	0.943±0.008	0.943±0.008	0.904±0.013	0.900±0.020	0.961±0.014	0.916±0.004
	EEGNet	0.946±0.005	0.946±0.005	0.908±0.008	0.916±0.015	0.958±0.003	0.964±0.004
	**Prop.**	0.949±0.008	0.949±0.008	0.913±0.013	0.907±0.021	0.967±0.019	0.952±0.008
**VS**	1D	0.941±0.007	0.941±0.006	0.923±0.008	0.923±0.020	0.952±0.018	0.945±0.003
	2D	0.935±0.014	0.935±0.014	0.916±0.018	0.928±0.023	0.939±0.013	0.940±0.006
	S.Fus	0.951±0.002	0.951±0.003	0.936±0.003	0.929±0.009	0.965±0.007	0.963±0.001
	EEGNet	0.962±0.005	0.963±0.004	0.952±0.006	0.955±0.013	0.967±0.006	0.971±0.003
	**Prop.**	0.974±0.005	0.974±0.004	0.956±0.008	0.957±0.013	0.982±0.005	0.976±0.004
**FL**	1D	0.968±0.007	0.968±0.007	0.956±0.009	0.966±0.010	0.968±0.007	0.947±0.003
	2D	0.935±0.008	0.934±0.009	0.906±0.014	0.876±0.040	0.968±0.017	0.939±0.003
	S.Fus	0.964±0.003	0.964±0.003	0.951±0.004	0.955±0.007	0.970±0.004	0.960±0.005
	EEGNet	0.955±0.007	0.955±0.007	0.938±0.009	0.951±0.008	0.957±0.011	0.963±0.005
	**Prop.**	0.977±0.003	0.977±0.003	0.960±0.006	0.961±0.015	0.984±0.005	0.978±0.002

On the Visual Oddball task, EEGNet came out ahead on sensitivity (0.916) and AUC-ROC (0.964), while the proposed model led on accuracy (0.949) and ADHD F1-score (0.913). No single architecture swept every metric on every task and this kind of task-level variation is expected given the different cognitive demands and trial structures across paradigms. The proposed model’s advantage is most consistent on the metrics most relevant to ADHD screening, namely accuracy and ADHD-class F1-score, across all four tasks.

EEGNet was a strong baseline, particularly on the Auditory Oddball sensitivity (0.887) and Visual Search AUC-ROC (0.971, essentially matching the proposed model at 0.976). This confirms that EEGNet remains competitive on single-paradigm EEG classification and the gains from the attention-based tri-stream fusion are most visible on overall accuracy, ADHD class F1-score and specificity, the metrics that most directly affect the false positive rate in a screening context. To check whether these architectural findings hold under stricter subject-independent conditions, we ran the full ablation under LOOCV on all four tasks; patient-level results are reported in S2 Table, where the proposed model with SES leads on accuracy and ADHD F1-score in every task.

### Subject-independent generalization (LOOCV)

To check how well the model holds up across completely unseen participants, we ran LOOCV on all four cognitive tasks. In each fold, one participant was held out as the test case while the model trained on everyone else. Every participant received exactly one majority-vote diagnosis, giving a clean patient-level evaluation that reflects a realistic screening scenario. Table 7 shows the results for both configurations across all four tasks.

**Table 7:** Patient-level LOOCV results across all four cognitive tasks. One majority-vote diagnosis per participant. Bold values mark the better-performing configuration per metric within each task. McNemar’s test compares With SES vs. Without SES paired patient predictions (Yates’ continuity correction; one-tailed).

Task	Config	Acc	Macro F1	ADHD F1	Sens	Spec	AUC	$\chi^{2}$	*p*
**AO** (*n* = 95)	Without SES	0.9053	0.8822	0.8302	0.7333	0.9846	0.9744	1.78	0.091
	With SES	**0.9579**	**0.9494**	**0.9286**	**0.8667**	**1.000**	**0.9836**		
**VO** (*n* = 84)	Without SES	0.9048	0.8886	0.8462	0.8462	0.9310	0.9683	1.13	0.144
	With SES	**0.9524**	**0.9454**	**0.9259**	**0.9615**	**0.9483**	**0.9874**		
**VS** (*n* = 59)	Without SES	0.8644	0.8347	0.7647	0.7647	0.9048	0.9216	0.44	0.252
	With SES	**0.9153**	**0.8906**	**0.8387**	**0.7647**	**0.9762**	**0.9776**		
**FL** (*n* = 60)	Without SES	0.8833	0.8408	0.7586	0.6471	0.9767	0.9549	0.17	0.342
	With SES	**0.9167**	**0.8863**	**0.8276**	**0.7059**	**1.000**	**0.9644**		

*Note. p*-values are one-tailed. Non-significance reflects the small number of discordant patient pairs per task (range: 6–9) rather than evidence of no effect. VS sensitivity is identical across configurations (0.7647) because the same 13 ADHD patients were correctly identified in both runs; the specificity gain (+7.1 pp) drives the overall accuracy improvement for that task. AUC = Area Under the ROC Curve, AO = Auditory Oddball; VO = Visual Oddball; VS = Visual Search; FL = Flanker.

Adding SES features pushed every single metric in the right direction on every task without exception. The accuracy gains at the patient level were +5.3 pp (Auditory Oddball), + 4.8 pp (Visual Oddball), + 5.1 pp (Visual Search) and +3.3 pp (Flanker). AUC-ROC gains were consistent across tasks: + 0.9 pp, + 1.9 pp, + 5.6 pp and +1.0 pp respectively. To test whether these gains were statistically meaningful at the patient level, we ran McNemar’s test on the paired patient predictions from both LOOCV runs. The Auditory Oddball result came closest to significance ($\chi^{2}=1.78$, *p* = 0.091, one-tailed), with 7 patients correctly flipping to the right answer when SES was added, against only 2 flipping the wrong way. The other three tasks did not reach *p* < 0.05 (Visual Oddball: *p* = 0.144; Visual Search: *p* = 0.252; Flanker: *p* = 0.342). This is a power problem rather than an absence of effect. Across all four tasks, only 6–9 patients changed their predicted diagnosis between the two runs and McNemar’s test simply cannot detect even large effects from so few discordant pairs. The consistent direction and the size of the accuracy gains suggest the SES benefit is real at the patient level, but confirming it statistically will need a larger multi-site study.

### External validation on an independent paediatric dataset

To provide preliminary evidence that the EEG backbone generalizes beyond our primary sample, we evaluated the dual-stream CNN-RNN architecture on an independent publicly available paediatric ADHD dataset [45]. This dataset comprises 19-channel EEG recordings from 61 children diagnosed with ADHD and 60 healthy controls (ages 7–12 years) completing a visual attention task. Relative to our primary adult sample, it differs in participant age and developmental stage, electrode array configuration (19-channel vs. 26-channel), cognitive task mechanics and the complete absence of socioeconomic metadata. Because no SES variables are available, only the EEG backbone (the “Without SES” configuration from our primary study) could be assessed here; the full SES-integrated model awaits validation on datasets that include socioeconomic information.

The architecture was applied without modification. The full 19-channel electrode set was used as input and all other pipeline components — 4-second non-overlapping epochs, preprocessing, training procedure and hyperparameters were held identical to the primary study. To allow direct comparison with the broad prior literature on this benchmark as well as with the subject-independent baseline of Sanchis et al. [60], we evaluated under two protocols: epoch-level cross-validation (both 5-fold and 10-fold) and subject-independent group 10-fold cross-validation, where participant identities are strictly separated between training and test folds.

Table 8 presents the results alongside prior studies. Under epoch-level validation the architecture achieves 97.5–97.9% accuracy, which is competitive with benchmarks on this dataset. Under the more stringent subject-independent protocol, accuracy falls to 80.4% (±6.4%), outperforming the subject-level baseline of Sanchis et al. (76.1%). The pronounced gap between epoch-level and subject-independent performance (approximately 17–18 percentage points) is consistent with the well-known optimism of epoch-level cross-validation in EEG studies and reinforces why subject-independent evaluation is the more clinically informative measure. These results suggest that the EEG backbone transfers to a paediatric population without architectural adaptation, while simultaneously confirming that subject-level generalization is the binding constraint on real-world performance.

**Table 8:** External validation on the Nasrabadi paediatric ADHD dataset [45] (*n* = 121; ages 7–12 yrs). Section A: prior epoch-level studies (benchmark context only; full metrics not reported in sources). Section B: our EEG backbone under epoch-level evaluation (= “Without SES” configuration). Section C: subject-independent evaluation with strict participant separation. SES pathway omitted — dataset contains no socioeconomic variables.

Study	Method	Protocol	Acc	Sens	Spec	W.F1
*A. Prior epoch-level studies*						
Mohammadi et al. [22]	Nonlinear EEG + MLP	70/10/20	0.937	—	—	—
Allahverdy et al. [61]	Nonlinear EEG + MLP	N/A	0.967	—	—	—
TaghiBeyglou et al. [51]	Raw EEG + CNN	5-fold	0.958	—	—	—
Talebi & Nasrabadi [62]	Connectivity + ANN	10-fold	0.991	—	—	—
Atila et al. [63]	Spiral + SVM	10-fold	0.975	—	—	—
Maniruzzaman et al. [52]	Time/morph. + GP	5-fold	0.975	—	—	—
Khare & Acharya [44]	VMD-HT + EBM	10-fold	0.998	—	—	—
Loh et al. [64]	CWT + CNN	10-fold	0.982	—	—	—
Tawhid et al. [30]	Spectrograms + CNN	5-fold	1.000	—	—	—
Ahire et al. [65]	DWT + NB	N/A	0.960	—	—	—
*B. Proposed EEG backbone — epoch-level* **^†^**						
**Proposed (5-fold)**	**Raw EEG^†^**	**5-fold**	0.975±0.064	0.982±0.076	0.969±0.060	0.976±0.071
**Proposed (10-fold)**	**Raw EEG^†^**	**10-fold**	0.979±0.010	0.983±0.016	0.973±0.020	0.979±0.014
*C. Subject-independent validation*						
Sanchis et al. [60]	EEG + CNN	Grp. 10-fold	0.761	0.855	—	0.807
**Proposed**	**Raw EEG^†^**	**Grp. 10-fold**	0.804±0.064	0.858±0.131	0.715±0.113	0.809±0.062

**^†^**Dual-stream CNN-RNN; full 19-channel input; pipeline unmodified from primary study. Section A results reproduced as reported; protocol differences preclude strict comparison. Large SD in Section B (5-fold) reflects small fold size (≈24 test samples). Grp.: group; NB: Naïve Bayes; N/A: not specified.

### Classification performance analysis

To assess whether the observed epoch-level benchmarking gains were consistent across folds, we examined fold-level score distributions via paired comparisons of the ‘With SES’ versus ‘Without SES’ configurations across all 20 metric categories (4 tasks × 5 metrics). Gains were directionally consistent across all folds in all 20 categories, with the majority of improvements on the Auditory and Visual Oddball tasks showing particularly stable fold-level differences. To formally assess the consistency of these fold-level differences while accounting for overlap between cross-validation training sets, we applied the **corrected resampled t-test** (Nadeau & Bengio, 2003 [66]), which explicitly adjusts the variance estimate by a factor of (1/k+1/(k−1))=0.45 for *k* = 5 folds, yielding a test that is ≈1.5× more conservative than the standard paired t-test. We additionally applied Bonferroni correction across all 20 simultaneous comparisons (adjusted threshold: α/m=0.05/20=0.0025) and Benjamini–Hochberg (BH) false discovery rate correction, which is more appropriate given the high intercorrelation among metrics within each task. Under the most conservative criterion (Bonferroni-corrected resampled test), 9 of 20 comparisons reached significance, covering the primary classification metrics of Accuracy, F1 and F1-ADHD across three of the four tasks (Auditory Oddball, Visual Search and Flanker). Under BH FDR correction, all 20 comparisons survived at α=0.05. Complete per-comparison statistics, including corrected t-values, Bonferroni-adjusted p-values, BH significance and 95% confidence intervals, are provided in S1 Table. However, the corrected resampled t-test adjusts for dependence arising from overlapping training sets across cross-validation folds but does not account for the non-independence of multiple epochs originating from the same subject. Accordingly, these epoch-level comparisons are interpreted as an exploratory assessment of the consistency of performance differences across folds and not as inferential evidence of an SES effect at the subject level. Subject-level inference was instead based on McNemar’s test applied to the paired LOOCV predictions from the ‘With SES’ and ‘Without SES’ configurations reported in Table 7. McNemar’s test did not reach statistical significance for any of the four tasks (*p* range: 0.091−−0.342). Thus, although the ‘With SES’ configuration showed consistently higher numerical *p*erformance across the reported LOOCV metrics, a statistically significant subject-level benefit of SES integration was not established in the present sample.

**Fig 4:**
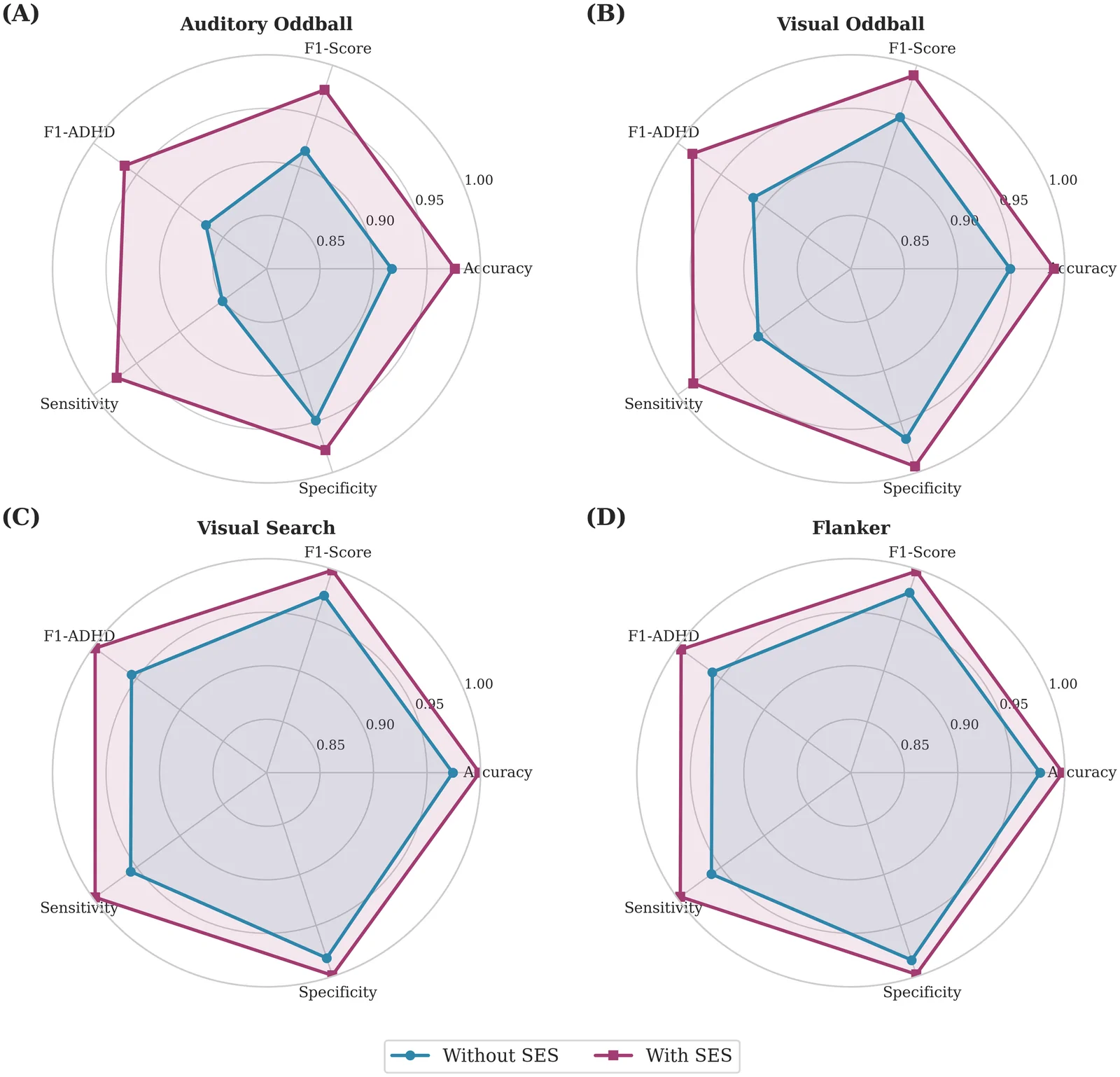
Task-specific radar charts: SES vs. no-SES across five metrics. Each axis represents one metric scaled from 0.80 to 1.00. (A) Auditory Oddball: the largest performance gap between configurations, most pronounced on the Sensitivity and F1-ADHD axes. (B) Visual Oddball: consistent improvement across all metrics. (C) Visual Search: near-ceiling performance for both configurations; the With SES polygon remains dominant. (D) Flanker: similar near-ceiling pattern with the With SES polygon fully enclosing the baseline. In all four panels, the With SES (filled, pink) polygon encloses the Without SES (filled, blue) polygon, confirming that the SES benefit holds at the individual task level rather than as an averaging artefact.

Fig 4 visualizes the task-level performance profile of each configuration across all five evaluation metrics. Panels (A)–(D) each show one cognitive task independently, preserving task-specific context. In every panel, the With SES polygon consistently encloses the Without SES polygon across all five metric axes, confirming that the SES-driven improvement is not an artefact of averaging across tasks with differing baseline levels. The most pronounced gap between configurations is visible in Fig 4A (Auditory Oddball), while Fig 4C and 4D (Visual Search and Flanker) show near-ceiling performance for both configurations, with the With SES polygon still clearly dominant.

Table 9 presents comprehensive 5-fold cross-validation results, showing consistent performance improvements with socioeconomic context integration. Visual search and flanker tasks achieved near-ceiling performance with SES integration, reaching 99.8–99.9% accuracy.

**Table 9:** Classification performance: Without vs. with SES. All values are means over 5 folds. F1-A = ADHD-class F1-score; AUC = area under the ROC curve; AO = Auditory Oddball; VO = Visual Oddball; VS = Visual Search; FL = Flanker. Difference rows show absolute percentage-point gains.

Task	Configuration	Acc	F1	F1-A	Sens	Spec	AUC
**AO**	Without SES	0.917	0.916	0.870	0.851	0.949	0.935
	With SES	0.976	0.976	0.964	0.973	0.978	0.976
	**Difference**	**+5.9**	**+6.0**	**+9.4**	**+12.2**	**+2.9**	**+4.1**
**VO**	Without SES	0.949	0.949	0.913	0.907	0.967	0.952
	With SES	0.990	0.990	0.983	0.982	0.994	0.984
	**Difference**	**+4.1**	**+4.1**	**+7.0**	**+7.5**	**+2.7**	**+3.2**
**VS**	Without SES	0.974	0.974	0.956	0.957	0.982	0.976
	With SES	0.999	0.999	0.998	0.998	0.999	0.994
	**Difference**	**+2.5**	**+2.5**	**+4.2**	**+4.1**	**+1.7**	**+1.8**
**FL**	Without SES	0.977	0.977	0.960	0.961	0.984	0.978
	With SES	0.998	0.998	0.996	0.997	0.998	0.994
	**Difference**	**+2.1**	**+2.1**	**+3.6**	**+3.6**	**+1.4**	**+1.6**

**Fig 5:**
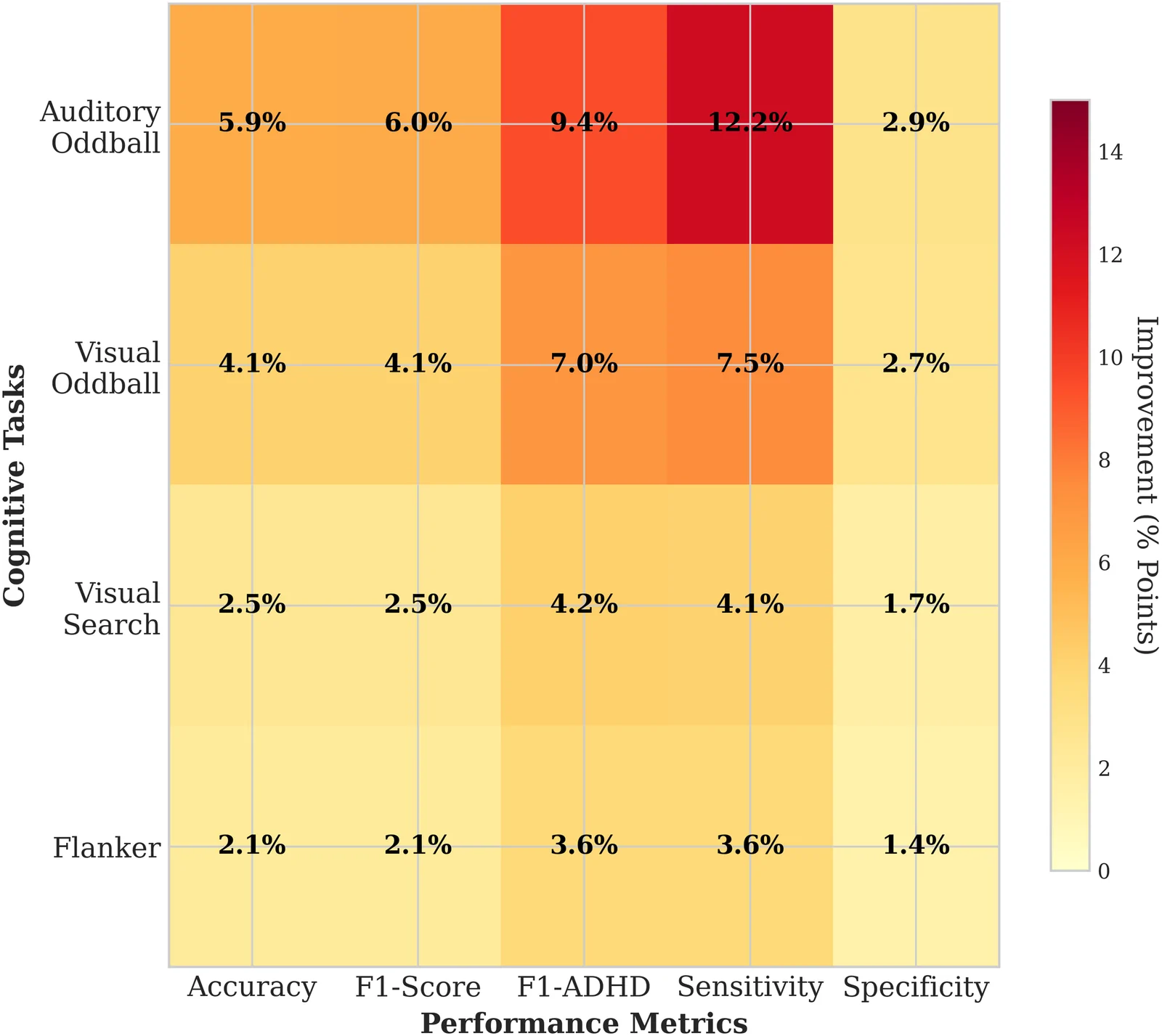
Heatmap of SES-driven performance improvements across tasks and metrics. Each cell shows the absolute percentage point gain (With SES minus Without SES) for a given task (rows) and evaluation metric (columns: Accuracy, Weighted F1, F1-ADHD, Sensitivity, Specificity). Warmer colors indicate larger gains. All cells are positive, confirming that SES integration improves every metric on every task. The auditory oddball task shows the largest overall gains, particularly on Sensitivity (+12.2 pp) and F1-ADHD (+9.4 pp), while visual search and flanker tasks show smaller but consistent improvements from a higher baseline.

Fig 5 quantifies the systematic improvements achieved through SES integration. The heatmap clearly shows that sensitivity and F1-ADHD metrics experience the largest gains across all tasks, with auditory oddball demonstrating the most substantial overall improvements (up to 12.2 percentage points).

**Fig 6:**
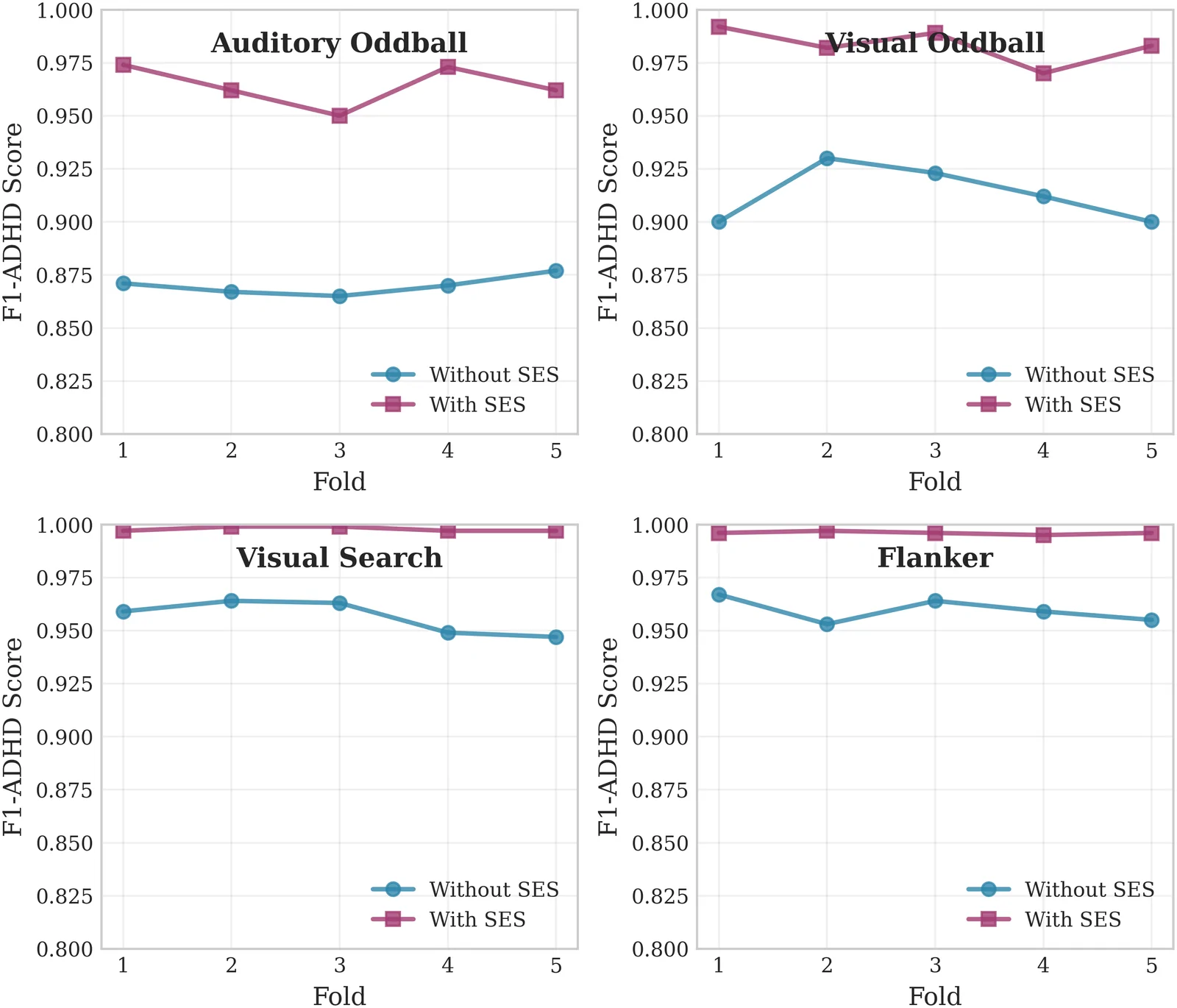
Cross-validation stability: F1-ADHD scores across five folds for all four cognitive tasks. Each panel shows fold-level F1-ADHD scores (y-axis, folds 1–5 on x-axis) for the Without SES (blue) and With SES (pink) configurations. In every panel and across every fold, the With SES line sits above the Without SES line, confirming that the performance gain is not driven by a single favorable fold but holds consistently across all data partitions. The narrow band of inter-fold variance (<1%) further indicates stable model behavior rather than sensitivity to the specific training split.

**Fig 7:**
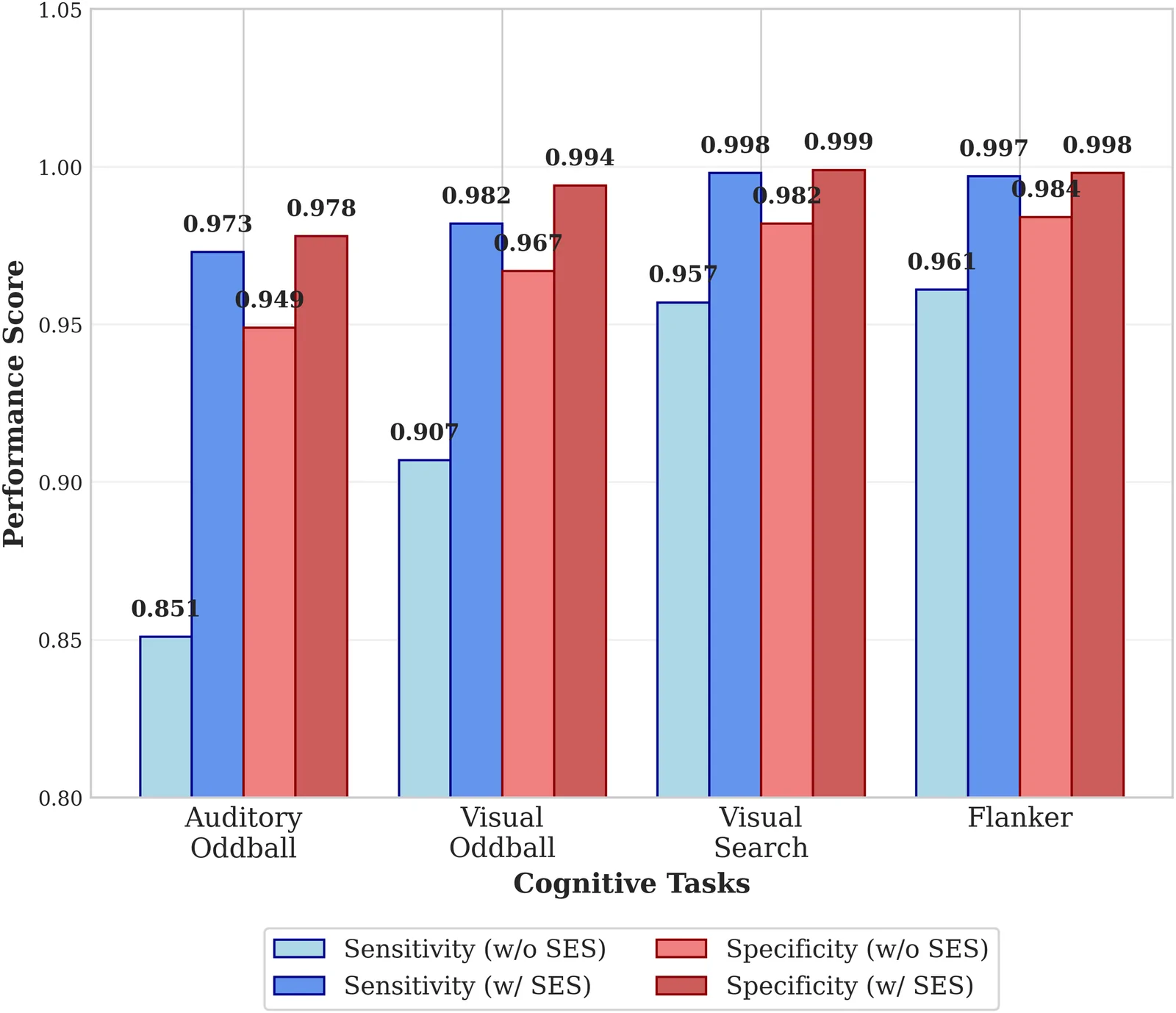
Class-wise performance: sensitivity and specificity across all four tasks and both model configurations. Grouped bars show Sensitivity (ADHD-screened-positive class) and Specificity (Control class) for the Without SES (blue) and With SES (pink) configurations, across all four cognitive tasks. SES integration consistently raises sensitivity while preserving or improving specificity, indicating that the multimodal model reduces missed screenings without introducing a corresponding rise in false positives, a balance that would be important in any real-world screening-support application.

Figs 6 and 7 provide critical insights into model reliability and clinical applicability. The consistent cross-validation performance (Fig 6) suggests reasonable generalization, while the balanced class-wise results (Fig 7) indicate the model’s ability to differentiate screened-positive from control participants across both sensitivity and specificity measures, which are characteristics important for any screening support tool.

### SHAP-based SES feature interpretability

**Fig 8:**
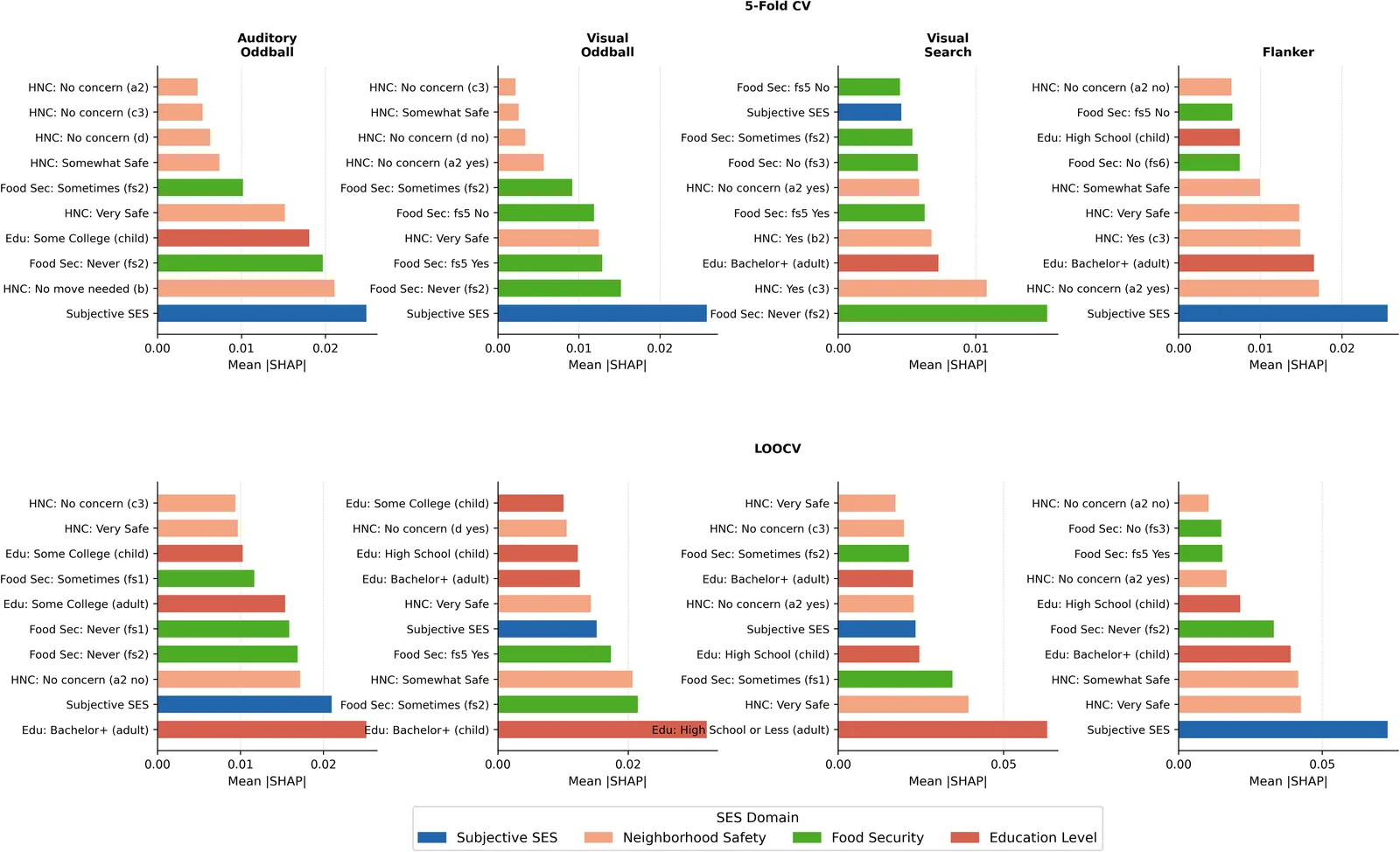
SHAP-based SES feature importance across cognitive tasks and validation protocols. Horizontal bars represent mean absolute SHAP values (marginal contribution to ADHD screening probability) for the top 10 SES features in each condition. Features are color-coded by SES domain: subjective standing (blue), neighborhood safety (orange), food security (green) and education level (red). Results are shown for both 5-fold cross-validation (top row) and leave-one-out cross-validation (bottom row) across all four cognitive tasks. The consistent ranking of the same SES domains across validation methods and tasks confirms the stability of these contributions rather than overfitting to a specific experimental condition.

To address the clinical need for model transparency, we applied SHAP (SHapley Additive exPlanations) [67] to quantify the marginal contribution of each SES feature to the model’s ADHD screening predictions. Because the SES sub-network processes tabular inputs independently of the EEG streams, we employed a KernelExplainer on the 40-dimensional SES input space, holding the EEG context fixed at the mean learned representation across all subjects. Separate analyses were conducted under both the 5-fold cross-validation and LOOCV protocols, yielding eight independent SHAP profiles (four tasks × two validation methods). Results are visualized in Fig 8.

Four SES domains emerged as the most consistent drivers of ADHD screening risk across all four cognitive tasks and both validation methods (Fig 8). *Subjective economic standing* (Subjective_SES) ranked as the top or near-top contributor in seven of eight profiles, with positive mean SHAP values indicating that lower perceived socioeconomic standing was associated with elevated ADHD screening risk, consistent with established socioeconomic gradients in ADHD prevalence [16]. *Neighborhood safety* features (hnc4_very safe, hnc4_somewhat safe) appeared in the top 15 features in six of eight profiles, with negative mean SHAP values suggesting that perceived neighborhood safety was associated with reduced screening risk. *Food security* indicators (fs2, fs5) appeared consistently across tasks in both validation protocols, reflecting the well-documented link between household economic instability and neurodevelopmental outcomes [16]. *Parental and child education level* also appeared in all eight profiles, with bachelor’s degree or higher education generally associated with lower screening risk.

The consistency of these rankings across tasks and validation methods, despite the SES sub-network comprising only 0.04% of total model parameters, suggests that these features provide stable, task-agnostic discriminative information rather than task-specific noise. It is important to note that SHAP values here reflect statistical associations within this screening dataset and should not be interpreted as causal pathways or clinical diagnostic criteria.

### Key findings

- **Consistent SES integration benefits.** Across all cognitive tasks, integrating socioeconomic features improved classification performance, with accuracy gains from 2.1% to 5.9% and particularly strong improvements in ADHD-class F1-scores (+3.6% to +9.4%).
- **Task-dependent performance patterns.** EEG-only baselines showed varying performance, with visual search and flanker (97.4%–97.7% accuracy) outperforming oddball tasks (91.7%–94.9%), suggesting cognitive control tasks yield more discriminative EEG patterns.
- **Balanced class performance.** The multimodal model maintained high performance on both classes, with sensitivity and specificity exceeding 97% across all tasks, which is important for screening applications where both false negatives and false positives carry practical costs.
- **Robust subject-independent generalization.** LOOCV demonstrated the model’s ability to generalize to entirely unseen participants. Across the four tasks, the With-SES configuration showed consistently higher numerical performance across the reported metrics than the Without-SES configuration. However, paired subject-level comparisons using McNemar’s test did not reach statistical significance for any task, and the observed SES-related improvements therefore require confirmation in larger cohorts.
- **Architectural validity and SOTA performance.** Ablation testing confirmed the necessity of the proposed attention-guided fusion mechanism, which outperformed single-modality streams, simple concatenation and a strong EEGNet benchmark.
- **Epoch-level benchmarking performance.** Under epoch-level cross-validation, visual search and flanker tasks achieved near-ceiling figures (>99.8% accuracy) with SES integration, reflecting strong architectural discrimination capacity within this evaluation regime. Subject-independent LOOCV results across all four tasks are reported in Table 7, providing the generalization-level estimates.
- **Preliminary cross-dataset generalization.** The EEG backbone, applied without modification to an independent paediatric dataset [45], achieved 80.4% accuracy under strict subject-independent evaluation, outperforming the subject-level baseline of Sanchis et al. [60] (76.1%) and confirming that the dual-stream architecture transfers across age groups and electrode configurations.

## Discussion

Our results suggest that integrating socioeconomic context with multimodal EEG representations can meaningfully improve ADHD risk screening, with consistent gains in accuracy (2.1–5.9%), ADHD-class F1-scores (3.6–9.4%) and sensitivity (3.6–12.2%) observed across diverse cognitive paradigms.

### Value of socioeconomic context

The consistent performance improvements suggest that socioeconomic information adds discriminative power beyond brain signals alone, translating established neuroscience on SES and brain development [15,16] into a working computational model. The particularly strong sensitivity improvements (up to 12.2%) suggest that socioeconomic variables capture variance in ADHD-related patterns that neurophysiological measures cannot fully represent on their own. Factors such as food insecurity or adverse home conditions may influence attention through pathways not fully reflected in EEG signatures, yet remain relevant to understanding individual symptom profiles.

The differential SES contribution across tasks is noteworthy. The auditory oddball task (passive attention) showed the largest improvements (+5.9%), while cognitive control tasks showed smaller but still meaningful gains (+2.1%). SHAP interpretability analysis identified subjective economic standing, neighbourhood safety perceptions and food security indicators as the most stable SES contributors across all four tasks and both validation protocols, offering an initial window into which environmental factors carry the most discriminative weight for risk screening.

These findings are consistent with a possible compensatory dynamic: socioeconomic context may be most informative when brain-based signals provide less discriminative certainty. One tentative interpretation is that the SES embedding helps the model resolve cases where neurophysiological signals are weaker or more ambiguous, as may occur in passive auditory paradigms. We acknowledge, however, that this remains a post-hoc interpretation and should not be taken as a mechanistic claim without dedicated explainability analyses.

### Architecture and clinical implications

Our attention-based fusion mechanism successfully integrated heterogeneous modalities, with 89.3% of parameters dedicated to fusion enabling adaptive, sample-specific weighting. The dual-stream EEG processing captured complementary temporal and spectro-temporal information, establishing strong EEG-only baselines (91.7–97.7%) upon which SES provided additional discriminative capacity. The compact SES sub-network (0.04% of parameters) demonstrates effective contextual integration without significant computational overhead.

From a practical standpoint, socioeconomic information collected via brief self-report questionnaires is low-cost and routinely available in many clinical and research settings, making integration into EEG-based screening workflows feasible without significant additional burden.

However, the equity implications of this approach require careful consideration. ADHD diagnosis rates vary systematically with SES [17], reflecting both genuine prevalence differences and well-documented disparities in diagnostic access. Because our screening labels derive from self-report rather than clinical diagnosis, they may already carry socioeconomic biases. A model trained on such labels risks learning to associate low SES with a screened-positive outcome in ways that encode, rather than correct, existing disparities. We therefore do not claim that this approach promotes health equity. Establishing whether SES integration is equitable would require dedicated fairness analyses (such as demographic parity and equal opportunity metrics across SES strata), which are beyond the scope of this proof-of-concept study and represent an important direction for future work.

The path from these proof-of-concept results to clinical translation requires considerable caution. Our binary classification is based on self-reported screening scores, which differ substantially from a comprehensive diagnostic evaluation. The high accuracy figures obtained under epoch-level cross-validation (97.6–99.9%) reflect within-dataset benchmarking rather than subject-independent performance; the corresponding LOOCV accuracies (91.5–95.8%) provide the more appropriate reference point when considering potential real-world screening performance. Even under this more conservative evaluation, these models should be understood as potential screening-support tools whose intended role is to flag individuals who may warrant a formal clinical referral, not to render a diagnostic decision or replace comprehensive clinical evaluation.

The two-tier validation framework maintains a clear distinction between architectural benchmarking and generalization claims. Epoch-level 5-fold cross-validation, conducted across all four tasks, provides a high-powered measure of the model’s discrimination capacity and is consistent with standard deep learning EEG benchmarking practice [30,51,52]. Because this protocol operates at the epoch level, these figures should be read as architectural performance estimates rather than subject-independent generalization figures. The LOOCV protocol, by contrast, guarantees complete participant separation and constitutes the primary basis for generalization claims. LOOCV results across all four tasks (Table 7) show that SES integration improved every reported metric on every task without exception, providing directionally consistent subject-independent evidence for the value of socioeconomic context.

McNemar’s test, the appropriate paired test for comparing With-SES and Without-SES predictions obtained from the same patients, did not reach statistical significance for any of the four tasks (*p* range: 0.091−−0.342). The small number of discordant prediction pairs (6–9 patients) limits the statistical power of these comparisons. Therefore, although SES integration was associated with consistently higher numerical performance across the evaluated LOOCV metrics in Table 7, the present results do not establish a statistically significant subject-level SES benefit. Larger, multi-site studies with greater sample sizes are needed to determine whether these observed improvements represent a reproducible subject-level effect.

To probe whether the EEG backbone generalizes beyond our primary sample, we evaluated the dual-stream CNN-RNN architecture on an independent paediatric dataset [45] without any architectural modification. Under subject-independent group 10-fold validation, the model achieved 80.4% accuracy, outperforming the subject-level baseline of Sanchis et al. [60] (76.1%), providing preliminary evidence that the architectural design is not tailored to a specific adult population or cognitive task set. Two important caveats apply. First, only the EEG backbone could be evaluated externally, as the Nasrabadi dataset contains no socioeconomic metadata; validating the full SES-integrated model on a dataset containing both EEG and SES information remains an important next step. Second, the substantial gap between epoch-level (97.5–97.9%) and subject-independent (80.4%) performance on this external dataset reinforces that epoch-level cross-validation is an optimistic estimator and that subject-level evaluation is the appropriate standard for assessing real-world transferability.

### Contributions and broader implications

Taken together, these findings point to four contributions. First, we provide proof-of-concept evidence that socioeconomic information carries quantifiable discriminative value for ADHD risk screening beyond neurophysiological signals alone. Second, the proposed attention-based fusion architecture offers a concrete mechanism for combining high-dimensional EEG streams with low-dimensional contextual vectors without letting one modality dominate the other. Third, the EEG backbone, applied without modification to an independent paediatric dataset [45], achieved 80.4% accuracy under strict subject-independent evaluation and outperformed the subject-level baseline of Sanchis et al. [60], providing preliminary evidence that the dual-stream design transfers across age groups and electrode configurations. Fourth, the consistency of SES-driven improvements across four cognitive paradigms suggests the effect is not task-specific, a prerequisite for any claim of practical generalizability.

More broadly, the consistent numerical differences observed between the With-SES and Without-SES configurations motivate further investigation of whether socioeconomic context may provide complementary information in neurophysiological classification models. Models incorporating both neural and contextual dimensions may offer a useful framework for studying the multifactorial nature of neurodevelopmental conditions, in line with prior work examining relationships between brain function and socioeconomic circumstances [16]. Brief socioeconomic questionnaires are already routinely collected in many clinical and research settings, potentially facilitating their evaluation alongside EEG-based screening approaches with minimal additional burden. However, the present study did not establish a statistically significant subject-level benefit of SES integration or a causal relationship between socioeconomic circumstances and neurophysiological function. These broader implications therefore require validation in larger independent clinical samples with gold-standard diagnostic criteria.

### Limitations and future directions

Several limitations warrant consideration, discussed in order of severity.

**External validation and sample generalizability.** These two concerns compound one another and are discussed together. Our dataset consists exclusively of university students (ages 18–30) with ADHD screening labels derived from self-report, which represents a substantial ecological mismatch with the clinical populations where a screening tool would ultimately be deployed. Clinically referred individuals typically present with more severe and persistent symptoms, greater psychiatric comorbidity and far greater socioeconomic heterogeneity than a university sample. Precisely because the sample is demographically narrow, the absence of external validation on a clinically recruited dataset is especially consequential: internal consistency on a homogeneous sample cannot be taken as evidence of generalizable performance. Preliminary external validation was conducted on the Nasrabadi paediatric dataset [45], but only the EEG backbone was tested, as that dataset contains no socioeconomic metadata. Validation of the full SES-integrated model on datasets containing both neurophysiological and socioeconomic data, drawn from clinically recruited samples with gold-standard diagnostic labels and greater demographic diversity, remains the most critical outstanding step. All within-dataset figures should be interpreted with this constraint in mind.

**Label validity.** The ADHD labels derive from a validated self-report screening instrument rather than formal clinical diagnoses. This is not merely a caveat about label quality; it is a fundamental boundary on what the system can claim to do. A participant who crosses the ASRS threshold may or may not meet criteria for ADHD under a structured clinical interview and the scale cannot distinguish true ADHD from conditions that share its symptom profile, including anxiety disorders, sleep disorders and learning disabilities. Every model in this paper, including all ablations and the external validation, is trained and evaluated on screening-level signal, not diagnostic certainty. The real-world value of this approach therefore depends on future validation against gold-standard clinical labels.

**Statistical power.** Subject-level LOOCV tests did not reach significance on any task (McNemar *p* range: 0.091−0.342). The core issue is statistical power rather than an absence of effect: only 6–9 subjects changed their predicted outcome when SES features were added, which is too few discordant pairs for McNemar’s test to detect even a genuine improvement. Larger, multi-site studies are needed to determine whether the SES benefit holds at the individual patient level.

**Recall bias.** Retrospective reports of childhood SES are inherently subject to recall bias, potentially introducing noise into the socioeconomic features.

**Interpretability constraints.** The compact SES sub-network design (0.04% of total parameters, 13,888 of 36.1M) inherently constrains individual feature-level SHAP magnitudes. A dedicated post-hoc interpretability analysis confirms that the relative ranking of SES contributors is stable across both validation protocols, offering actionable insight into which socioeconomic domains drive model decisions. Deeper interpretability work remains a priority for future research.

**Hyperparameter optimization.** The substantial computational footprint of the tri-stream architecture precluded exhaustive automated hyperparameter search, suggesting marginal gains may still be achievable through more systematic optimization.

**Confounding variables.** We did not account for potentially confounding factors such as sleep quality, medication status, or psychiatric comorbidities.

Future research should prioritize cross-dataset validation with gold-standard diagnostic labels, mechanistic investigations using mediation analysis, extension to other neurodevelopmental conditions and prospective clinical trials assessing real-world screening utility. Future work should extend interpretability beyond the SES pathway by applying Grad-CAM to the EEG streams, which would spatially map which electrode regions and frequency bands drive model decisions and reveal how neurophysiological and socioeconomic signals interact at the feature level.

## Conclusion

This study provides proof-of-concept evidence for a framework that integrates socioeconomic context with multimodal neurophysiological data for ADHD risk screening. Under subject-independent Leave-One-Subject-Out Cross-Validation, the more conservative evaluation tier, adding SES features raised accuracy from 90.5% to 95.8% on Auditory Oddball, 90.5% to 95.2% on Visual Oddball, 88.3% to 91.7% on Flanker, and 86.4% to 91.5% on Visual Search. The direction of improvement was consistent across the reported metrics and tasks, although McNemar’s test on paired patient predictions did not reach statistical significance on any task. Under 5-fold stratified cross-validation, an epoch-level architectural benchmarking tier, the same comparison yielded larger accuracy improvements (2.1%–5.9%) and sensitivity gains (up to 12.2%) across the four cognitive paradigms; these figures should be interpreted as within-dataset benchmarking results rather than estimates of subject-independent generalization. Rigorous ablation studies further supported the architectural design choices, with the proposed model outperforming single-stream baselines and a strong EEGNet benchmark.

As discussed above, the contributions span four areas: an evaluation of the potential incremental value of SES alongside EEG-based features, an attention-based fusion architecture designed to prevent modality imbalance, preliminary cross-dataset generalization to a paediatric sample [45] that outperforms prior subject-level benchmarks [60], and consistent numerical SES-related performance differences across all four cognitive tasks under both evaluation tiers.

Beyond technical performance, this approach challenges the prevailing paradigm of developing neurophysiological biomarkers in isolation from environmental realities. Brain function develops and operates within socioeconomic circumstances that shape both neurodevelopment and symptom expression. While this proof-of-concept requires future validation in diverse clinical populations using gold-standard diagnostic criteria and XAI feature mapping, the foundational results are encouraging. Incorporating readily available contextual data alongside neurophysiological measurements represents a promising step toward more accurate, equitable and comprehensive screening tools.

## Supporting information

S1 TableFull statistical comparison results: With SES vs. Without SES across all 20 task–metric pairs.Corrected resampled t-statistics and p-values use the Nadeau–Bengio (2003) correction factor (1/k+1/(k−1))=0.45 for *k* = 5 folds. Bonferroni threshold: α/m=0.05/20=0.0025. BH = Benjamini–Hochberg FDR correction. CI = 95% confidence interval on the mean difference. The corrected resampled *t*-test accounts for dependence arising from overlapping training sets across cross-validation folds but does no*t* account for within-subject dependence among epochs. These epoch-level comparisons are therefore reported as exploratory assessments of the consistency of performance differences across folds and are not used to establish a subject-level effect of SES integration. Task abbreviations: AO = Auditory Oddball; VO = Visual Oddball; VS = Visual Search; FL = Flanker. W/o SES = Without SES; W/ SES = With SES. ^***^Significant after Bonferroni correction (*p*_Bonf_ < 0.05). ^†^Not significant after Bonferroni correction, but significant under BH FDR correction at α=0.05. All values are means over 5 folds; corrected t-test *df* = 4 for all comparisons.(PDF)

S2 TableLOOCV ablation and baseline comparison: Patient-level results.One majority-vote diagnosis per participant; no ± std reported (LOOCV produces a single evaluation per configuration). Bold values mark the best result per metric within each task. Model abbreviations: 1D = 1D Stream Only; 2D = 2D Stream Only; S.Fus = Simple Fusion; EEGNet = EEGNet (SOTA baseline); Prop. = Proposed model without SES; Prop. + SES = Proposed model with SES integration. Task abbreviations: AO = Auditory Oddball; VO = Visual Oddball; VS = Visual Search; FL = Flanker. EEGNet shows higher sensitivity than the proposed model on Visual Search and Flanker, reflecting a different sensitivity–specificity trade-off rather than overall superiority; the proposed model leads on Accuracy, ADHD F1 and AUC on both tasks.(PDF)
