# Supplementary material for: Integrating socioeconomic context with multimodal EEG data for improved ADHD risk screening
Source: PLoS One. 2026 Sep 1;21(9):e0357213. doi: 10.1371/journal.pone.0357213 (PMC13533352; doi:10.1371/journal.pone.0357213)
Supplement: Supplementary file 1 [file pone.0357213.s001.pdf]

S1 Table. Full statistical comparison results: With SES vs. Without SES across all 20 task–metric pairs. Corrected resampled t-statistics and p-values use the Nadeau–Bengio (2003) correction factor $(1/k + 1/(k-1)) = 0.45$ for $k = 5$ folds. Bonferroni threshold: $\alpha/m = 0.05/20 = 0.0025$. BH = Benjamini–Hochberg FDR correction. CI = 95% confidence interval on the mean difference. The corrected resampled t-test accounts for dependence arising from overlapping training sets across cross-validation folds but does not account for within-subject dependence among epochs. These epoch-level comparisons are therefore reported as exploratory assessments of the consistency of performance differences across folds and are not used to establish a subject-level effect of SES integration. Bold marks the largest mean difference and the most significant comparison within each task.

Task	Metric	W/o SES	W/ SES	Mean Diff	95% CI	$t_{\mathbf{corr}}$	$p_{\mathbf{Bonf}}$	BH Sig.
**AO**	Accuracy	0.9168	0.9764	+0.0596	[0.0545, 0.0647]	**22.71**	**0.000445*****	Yes
	F1	0.9160	0.9762	+0.0602	[0.0554, 0.0650]	20.22	0.000706***	Yes
	F1-ADHD	0.8700	0.9642	+0.0942	[0.0848, 0.1036]	15.58	0.001980***	Yes
	Sensitivity	0.8508	0.9732	**+0.1224**	[0.0714, 0.1734]	5.18	0.132†	Yes
	Specificity	0.9488	0.9780	+0.0292	[0.0010, 0.0574]	3.26	0.624†	Yes
**VO**	Accuracy	0.9492	0.9900	+0.0408	[0.0243, 0.0573]	6.30	0.065†	Yes
	F1	0.9488	0.9900	+0.0412	[0.0249, 0.0575]	**6.38**	**0.062**†	Yes
	F1-ADHD	0.9130	0.9832	+0.0702	[0.0395, 0.1009]	6.21	0.069†	Yes
	Sensitivity	0.9068	0.9822	**+0.0754**	[0.0382, 0.1126]	5.17	0.133†	Yes
	Specificity	0.9668	0.9936	+0.0268	[0.0103, 0.0433]	3.98	0.328†	Yes
**VS**	Accuracy	0.9744	0.9988	+0.0244	[0.0163, 0.0325]	8.59	0.020***	Yes
	F1	0.9744	0.9988	+0.0244	[0.0163, 0.0325]	8.59	0.020***	Yes
	F1-ADHD	0.9564	0.9978	**+0.0414**	[0.0281, 0.0547]	**8.75**	**0.019*****	Yes
	Sensitivity	0.9566	0.9976	+0.0410	[0.0218, 0.0602]	5.41	0.113†	Yes
	Specificity	0.9816	0.9992	+0.0176	[0.0091, 0.0261]	5.90	0.083†	Yes
**FL**	Accuracy	0.9772	0.9978	+0.0206	[0.0152, 0.0260]	**9.34**	**0.015*****	Yes
	F1	0.9772	0.9978	+0.0206	[0.0152, 0.0260]	**9.34**	**0.015*****	Yes
	F1-ADHD	0.9596	0.9960	**+0.0364**	[0.0256, 0.0472]	8.77	0.019***	Yes
	Sensitivity	0.9612	0.9970	+0.0358	[0.0010, 0.0706]	3.32	0.587†	Yes
	Specificity	0.9836	0.9980	+0.0144	[0.0044, 0.0244]	4.11	0.295†	Yes

***Significant after Bonferroni correction ($p_{\mathrm{Bonf}} < 0.05$). †Not significant after Bonferroni correction, but significant under BH FDR correction at $\alpha = 0.05$. All values are means over 5 folds. Corrected t-test $df = 4$ for all comparisons. AO = Auditory Oddball; VO = Visual Oddball; VS = Visual Search; FL = Flanker; W/o SES = Without SES; W/ SES = With SES.
