# Supplementary material for: Integrating socioeconomic context with multimodal EEG data for improved ADHD risk screening
Source: PLoS One. 2026 Sep 1;21(9):e0357213. doi: 10.1371/journal.pone.0357213 (PMC13533352; doi:10.1371/journal.pone.0357213)
Supplement: Supplementary file 2 [file pone.0357213.s002.pdf]

S2 Table. LOOCV ablation and baseline comparison: patient-level results. One majority-vote diagnosis per participant; no ± std reported (LOOCV produces a single evaluation per configuration). Bold values mark the best result per metric within each task. Model abbreviations: 1D = 1D Stream Only; 2D = 2D Stream Only; S.Fus = Simple Fusion; EEGNet = EEGNet (SOTA baseline); Prop. = Proposed model (without SES); Prop.+SES = Proposed model with SES integration. Task abbreviations: AO = Auditory Oddball; VO = Visual Oddball; VS = Visual Search; FL = Flanker. Macro F1 = macro-averaged F1-score; ADHD F1 = ADHD-class F1-score; AUC = area under the ROC curve.

Task	Model	Accuracy	Macro F1	ADHD F1	Sensitivity	Specificity	AUC
**AO**	1D	0.9474	0.9373	0.9123	0.8667	0.9846	**0.9969**
	2D	0.8947	0.8855	0.8529	**0.9667**	0.8615	0.9728
	S.Fus	0.9368	0.9224	0.8889	0.8000	**1.000**	0.9903
	EEGNet	0.8526	0.8263	0.7586	0.7333	0.9077	0.9649
	Prop.	0.9053	0.8822	0.8302	0.7333	0.9846	0.9744
	**Prop.+SES**	**0.9579**	**0.9494**	**0.9286**	0.8667	**1.000**	0.9836
**VO**	1D	0.8690	0.8375	0.7660	0.6923	0.9483	0.9635
	2D	0.8452	0.8349	0.7937	**0.9615**	0.7931	0.9615
	S.Fus	0.8690	0.8451	0.7843	0.7692	0.9138	0.9668
	EEGNet	0.8690	0.8281	0.7442	0.6154	**0.9828**	0.9562
	Prop.	0.9048	0.8886	0.8462	0.8462	0.9310	0.9683
	**Prop.+SES**	**0.9524**	**0.9454**	**0.9259**	**0.9615**	0.9483	**0.9874**
**VS**	1D	0.8644	0.8127	0.7143	0.5882	**0.9762**	0.9762
	2D	0.8644	0.8212	0.7333	0.6471	0.9524	0.9594
	S.Fus	0.8475	0.7943	0.6897	0.5882	0.9524	0.9314
	EEGNet	0.8644	0.8447	0.7895	**0.8824**	0.8571	0.9398
	Prop.	0.8644	0.8347	0.7647	0.7647	0.9048	0.9216
	**Prop.+SES**	**0.9153**	**0.8906**	**0.8387**	0.7647	**0.9762**	**0.9776**
**FL**	1D	0.8333	0.7671	0.6429	0.5294	0.9535	0.9549
	2D	0.8667	0.8137	0.7143	0.5882	0.9767	0.9603
	S.Fus	0.9000	0.8602	0.7857	0.6471	**1.000**	0.9576
	EEGNet	0.8500	0.8291	0.7692	**0.8824**	0.8372	0.9015
	Prop.	0.8833	0.8408	0.7586	0.6471	0.9767	0.9549
	**Prop.+SES**	**0.9167**	**0.8863**	**0.8276**	0.7059	**1.000**	**0.9644**

Note. EEGNet shows higher sensitivity than the proposed model on Visual Search and Flanker, reflecting a different sensitivity–specificity trade-off rather than overall superiority; the proposed model leads on Accuracy, ADHD F1, and AUC on both tasks.
